# Links between chronic health conditions and sexual dysfunction in men: insights from a German population-based study

**DOI:** 10.1186/s12894-026-02416-7

**Published:** 2026-11-05

**Authors:** Selina Marie Kronthaler, Carlotta Florentine Oesterling, Tatjana Tissen-Diabaté, Klaus Michael Beier, Thorsten Schlomm, Jörg Neymeyer, Laura Hatzler

**Affiliations:** 1 Institute of Sexology and Sexual Medicine, Charité – Universitätsmedizin Berlin, Berlin, Germany; 2 Institute of Social Medicine, Epidemiology and Health Economics, Charité – Universitätsmedizin Berlin, Berlin, Germany; 3 Department of Urology, Charité – Universitätsmedizin Berlin, Berlin, Germany

**Keywords:** Chronic health conditions, Sexual dysfunction, Erectile dysfunction, Male sexual dysfunction, Cardiovascular disease, Sexual health

## Abstract

**Background:**

Sexual dysfunction (SD) is common among men and significantly impacts quality of life. Chronic health conditions (CHC), particularly with comorbid mental health (MH) issues, may be associated with increased risk of SD, yet large-scale population data are limited.

**Objective:**

This study examined the prevalence and associations of sexual problems (SP), ICD-11-defined SD, and sexual distress among men with and without CHC in a representative German sample.

**Methods:**

From a cross-sectional online survey, data from *N =* 1742 cisgender men (unweighted sample size) were included in the analysis. Participants reported on CHC status (i.e., cardiovascular, urological, mental health, cancer), SP, SD, and sexual distress (SDS-DAO). Regression models assessed associations between CHC and SD. Network analyses explored interrelations between CHC subgroups and SD domains.

**Results:**

CHC were reported by 50.8% of participants. Men with CHC had higher rates of SP (79.2%) and SD (23.2%) than those without CHC (66.7% SP, OR 1.90, 95% CI 1.52–2.37; 9.1% SD, OR 3.00, 95% CI 2.24–4.07). SD was most prevalent in men with urological disorders, cancer, pain-related, mental health and cardiovascular conditions. Among men with CHC, comorbid MH conditions were substantially associated with sexual distress (β = 3.4, 95% CI 1.3–5.4, *p* = .001*).* Regression models demonstrated strong associations between CHC, comorbid MH conditions, and SD, with network analysis revealing strong links between erectile dysfunction with hypoactive sexual desire dysfunction and delayed ejaculation dysfunction, and conditions such as urological disorders, mental health conditions, cancer and cardiovascular disease.

**Conclusion:**

CHC, especially mental health conditions, urological disorders, cancer and cardiovascualr disease, are strongly associated with SD and sexual distress in men. Integrated care approaches that include routine sexual health screening and address mental wellbeing may thus be essential to improving outcomes for men with chronic illness.

**Supplementary Information:**

The online version contains supplementary material available at https://doi.org/10.1186/s12894-026-02416-7.

## Introduction

Sexual health comprises a fundamental component of overall health and wellbeing [1]. However, with a 12-month prevalence of 45.7%, a substantial proportion of adults report sexual problems (SP) regarding desire, arousal, orgasm, or ejaculation [2]. A wide array of studies on SP are based on measures of sexual function [3, 4]. However, beyond physical satisfaction and reproduction, sexuality also serves as a source of connection and intimacy [5]. As sexual health is thus an important aspect of quality of life [6], SP frequently elicit significant sexual distress. The International Classification of Diseases (11th revision; ICD-11 [7]) defines a SP concomitant to sexual distress as “sexual dysfunction” (SD), provided that it persists for at least 6 months. ICD-11 conceptualizes sexual dysfunctions as multifactorial conditions resulting from the interaction of physiological, psychological, interpersonal, social, and cultural factors. Accordingly, sexual dysfunctions may occur in association with medical conditions, mental health conditions, medications, and other etiological factors [7]. The 12-month prevalence of SD according to ICD-11 criteria is 16.5% in the general population and 13.3% in men [2]. SD may manifest as erectile dysfunction, premature ejaculation, hypoactive sexual desire, or orgasmic dysfunction [2]. A nationwide survey study on sexual health found 6.6% of sexually active men in Germany to be affected by erectile dysfunction (ED), 4.5% by premature ejaculation, 3.3% by hypoactive sexual desire dysfunction, and 3.1% by orgasmic dysfunction [8]. Reported prevalence rates differ between age groups and comorbid conditions [2, 9]. Although sexual distress is a required criterion for diagnosis of SD according to ICD-11 guidelines, sexual distress remains frequently unaddressed in male SD research [3, 4].

One factor associated with SP and SD is the presence and treatment of chronic health conditions (CHC). It is estimated that over 60% of the general population in Germany is affected by at least one CHC [10]. While recent advances in CHC treatment result in improvements in QoL and survival rates of people with CHC, CHC themselves – along with the side effects and complications associated with surgical or medical treatment interventions – may adversely impact physical and, thus, sexual functioning [11]. Further, the management of CHC in daily life may pose significant challenges, thereby augmenting distress and diminishing overall wellbeing [12–15]. The impact of CHC on sexual desire, arousal, genital response, or orgasmic functioning may be direct or indirect. Direct effects may emanate from vascular dysfunction, endocrine imbalances (e.g., testosterone deficiency), neuropathy, and neurobiological alterations that are elicited by medication, surgery, or radiation [16–20]. Indirect effects may occur via changes in self-image, mood, mobility, and energy, relationship difficulties, and/or fears and worries surrounding sexuality and fertility [21].

While observed rates of SD in men with CHC are significantly higher than in the general population, most clinical studies focus on samples with erectile dysfunction as SD outcome variable. The association between ED and cardiovascular and metabolic conditions (e.g., cardiovascular disease, atherosclerosis, hypertension, diabetes, dyslipidemia, osteoporosis) is well established and has given rise to a plethora of research and clinical interventions [22, 23]. Shared pathophysiological processes such as atherosclerosis yield ED as a predictive marker of future cardiovascular events [22]. Both cardiovascular disease and ED have been associated with endothelial dysfunction, inflammation, diabetes, and low plasma testosterone levels as common risk factors [22] and more than 70% of men with cardiovascular diseases experience ED. Besides cardiovascular conditions, other CHC have been associated with ED. For example, a large-scale clinical cohort study found 38.8% of men with ED above 18 years to have at least one CHC [24].

Given the strong focus on clinical populations diagnosed with individual CHC and the marked emphasis on ED, the association between disjoint CHC and SD remains insufficiently explored in large, population-based samples. Although less well established, SDs have further been associated with the diagnosis and treatment of urological conditions (i.e., lichen sclerosus, urinary tract infections, incontinence, pelvic floor dysfunction [25]; infectious, inflammatory, or autoimmune diseases (i.e., psoriasis, joint inflammation, rheumatoid arthritis, rheumatism, sexually transmitted infections, HIV/AIDS [9]; pain related conditions (i.e., chronic pain, chronic pelvic pain, bladder pain syndrome [26, 27]); neurological conditions (i.e., Alzheimer’s disease, dementia, epilepsy, multiple sclerosis, Parkinson’s disease, stroke, cerebral palsy [28–30]); and cancer in pelvic and other regions [31]. Despite this growing body of evidence, population-based studies examining SD across multiple CHC types simultaneously – and accounting for comorbid mental health conditions – remain limited.

In individuals with mental health conditions (e.g., major depressive disorder, anxiety disorders, post-traumatic stress disorder), SD prevalence rates of up to 65% have been reported [32] – with notable variations across demographic and age groups [33–38]. In light of the substantial impact of CHC on quality of life and wellbeing, comorbid mental health conditions may constitute a potential underlying factor associated with both CHCs and SDs [39, 40]. Symptoms commonly associated with internalizing disorders (e.g., depression and anxiety), such as anhedonia, diminished motivation, and cognitive disengagement, may contribute to or exacerbate SP and SD [41]. Moreover, although the association between mental health conditions and SD appears to persist independently of antidepressant or psychotropic medication use, these medications have also been widely reported to produce adverse effects on sexual functioning, including desire, arousal, and orgasm [42, 43].

SD domains are typically examined individually, which limits direct comparisons of domain-specific prevalence rates across SD samples. As outlined above, CHC can affect both somatic and mental health [16–21]. The World Health Organization defines mental health as “a state of mental well-being that enables people to cope with the stresses of life, realize their abilities, learn and work well, and contribute to their community” [44]. Despite the strong association between mental health conditions and sexual function and the existing evidence on shared underlying risk factors [39, 40], comorbid mental health conditions are frequently disregarded when examining individual somatic CHC and SD and respective treatment options.

The present study aims to assess and compare the prevalence of self-reported SP, SD, and sexual distress among men with and without CHC. The present study also aims to compare these same 3 outcomes among men with CHC based on the presence or absence of mental health conditions. To address existing research gaps, the study also examines prevalence differences of self-reported SP, SD, and sexual distress across specific CHC subgroups, including mental health, cardiovascular and metabolic, infectious, inflammatory, cancer-related, pain-related, and neurological conditions. Furthermore, it investigates whether the presence of CHC, co-occurring mental health conditions, or specific CHC types are associated with an increased likelihood of experiencing SD and sexual distress. By offering a more differentiated understanding of how various CHCs relate to men’s sexual health, the study aims to inform future research, prevention, and intervention strategies.

## Methods

### Procedure

This cross-sectional survey study is based on a sample representative of the German population in terms of gender, age, and federal state [45]. Participants were recruited in December 2021 via YouGov Deutschland, an independent research and analytics institute, as part of a research project funded by a Patient and Stakeholder Engagement (PSE) grant. The survey was developed in collaboration with patient organizations to explore help-seeking behaviors and healthcare needs related to sexual problems in Germany.

As part of the PSE-funded project, eight individuals with personal or professional experience of SP were actively involved in patient and public involvement activities. Their contributions informed both the co-development of questionnaire items and the selection of validated instruments, with the aim of improving relevance and reducing non-response bias.

In addition to sociodemographic and health-related variables already recorded by YouGov, the final instrument included items on sexual outcomes, self-reported received diagnoses, biopsychosocial protective and risk factors, help-seeking behavior, and healthcare needs. Further details on stakeholder involvement, questionnaire development, the specific items assessed, and the validated instruments used are provided in a separate publication focusing on CHC linked to SD in women [46].

This study adheres to the STROBE guidelines for cross-sectional studies and received ethical approval from the Ethics Committee of Charité – Universitätsmedizin Berlin (EA4/221/21).

### Participants

**Fig. 1:**
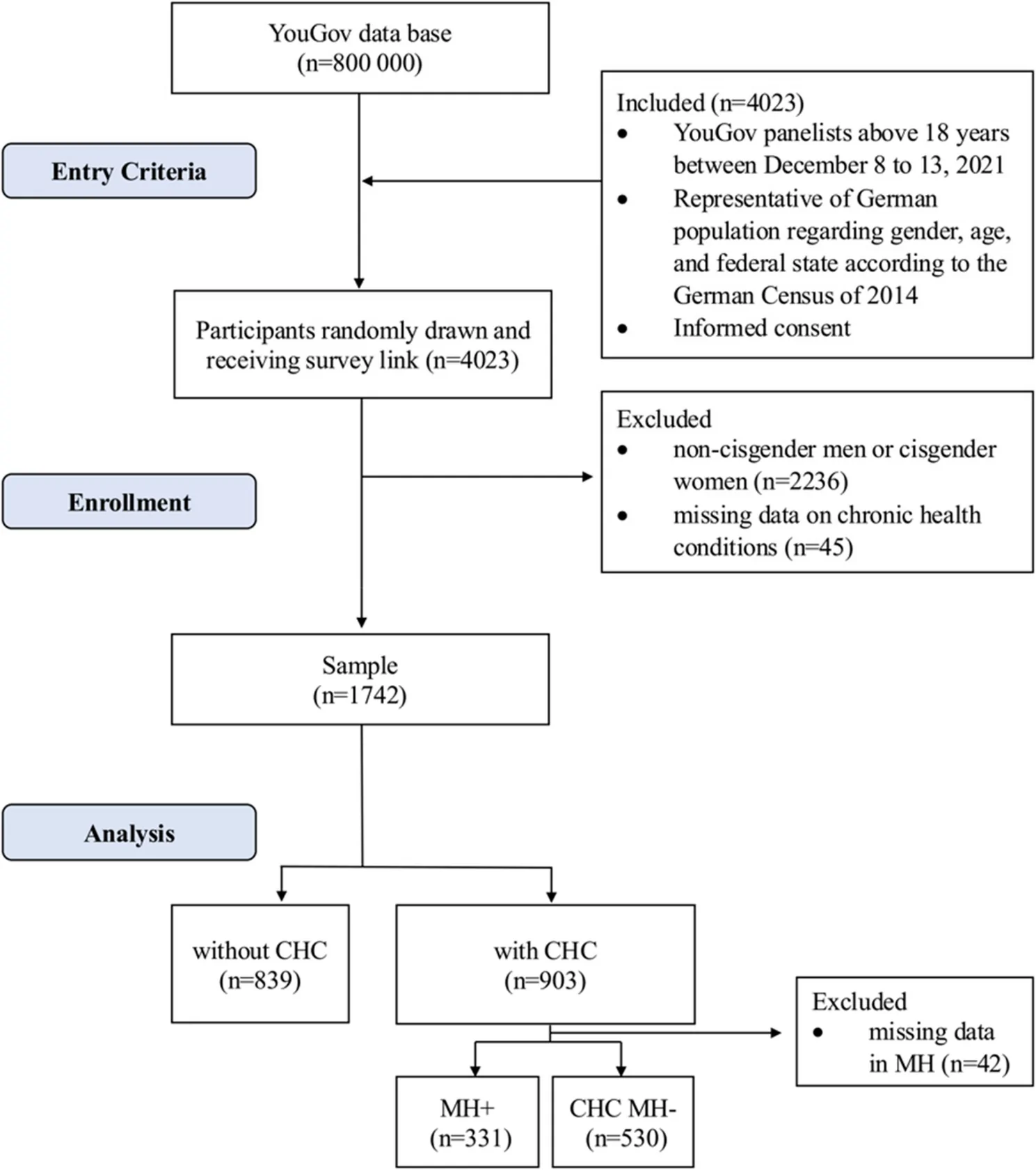
Flow Chart of Recruitment Procedure. Unweighted frequencies. Note. CHC: chronic health condition(s). MH: mental health CHC. MH+: CHC with at least one comorbid mental health condition. CHC MH–: CHC without any comorbid mental health condition

A random sample was drawn from the YouGov Germany panel of 800 000 individuals, using YouGov’s proprietary “turbo sampling” method. This approach continuously selects small random subsamples across active surveys, dynamically balancing completion rates within predefined quota categories (age, sex, and federal state) to ensure representativeness. Quotas were dynamically adjusted during fieldwork to ensure representativeness across demographic strata. The survey was distributed to adult panelists until the targeted sample size of *N =* 4000 was reached. This sample size was determined a priori based on feasibility and budget considerations to ensure sufficient precision for population-level estimates. For a Flowchart of the recruitment procedure, see Fig. 1. Among 4430 invited panelists, 4122 completed the survey (93% completion). After excluding 99 participants (2.4%) who were flagged during a standard data-cleaning process for internally inconsistent demographic responses (e.g., contradictory age or sex entries), the final analytical sample comprised 4023 respondents. By applying survey weights, the data was adjusted to accurately reflect the German population in terms of age, sex, and geographic distribution [46]. Participants received a compensation of 500 YouGov points (equivalent to 1€) for completing the 20-minute questionnaire. All questions could be skipped without penalty. For further details on the sampling process, see the concordant publication on CHC linked to SD in women [46]. A total of *N =* 4023 adults completed the questionnaire, of which *N =* 1787 cisgender men (hereafter referred to as men) were included in the present study. The analysis of the female sample (*N =* 2016) was published elsewhere [48] and non-binary respondents (*n =* 220) will be subject to future analyses by the author team. Of all men, *n =* 45 were excluded due to missing CHC status, resulting in *N =* 1742 men being subject of further analyses. The mean age was *M* = 49.6 years (*SD* = 16.9). Further demographic characteristics are presented in Table 1, including relationship status (61.2% in a relationship) and sexual orientation (88.9% heterosexual, 4.2% homosexual, and 4.7% bisexual.

#### CHC Subgroups

Participants were first divided into two groups based on the presence or absence of at least one CHC, yielding a dichotomous CHC status variable (CHC status: yes vs. no). Within the CHC group, participants were further subdivided according to whether their CHC(s) were exclusively somatic or included at least one comorbid mental health condition, yielding a dichotomous MH status variable with two subgroups: CHC MH– (somatic CHC only) and CHC MH+ (CHC with comorbid mental health condition). In addition, each specific CHC category was examined using two overlapping classifications: a “disjoint” subgroup, comprising participants who reported only that specific CHC without any other comorbid CHC, and a “comorbid” subgroup, comprising all participants who reported that specific CHC regardless of whether additional CHC were present. The specific CHC categories examined were: mental health (i.e., depression, anxiety, post-traumatic stress disorder, autism, other mental health conditions), cardiovascular and metabolic (i.e., cardiovascular disease, atherosclerosis, hypertension, diabetes, dyslipidemia, osteoporosis), urological conditions (i.e., lichen sclerosus, urinary tract infections, incontinence, pelvic floor dysfunction, infertility for more than 6 months), infection or inflammation (i.e., psoriasis, joint inflammation, rheumatoid arthritis, rheumatism, sexually transmitted infections, HIV/AIDS), cancer (i.e., breast, prostate, other), pain (i.e., chronic pain, chronic pelvic pain, bladder pain syndrome), and neurological disease (i.e., Alzheimer’s disease, dementia, epilepsy, multiple sclerosis, Parkinson’s disease, stroke, cerebral palsy).

### Measures

#### Demographic variables

Sociodemographic variables included age in years (further grouped into age categories), monthly net income (<€2,500, €2,500–5,000, >€5,000), and sexual orientation (heterosexual, homosexual, bisexual, other/prefer not to disclose). The following variables were assessed as binary (yes/no): education level (> 12 years), employment status, religious identification, current relationship status, migration background (self or parental immigration after 1949), presence of at least one child in the household, household size of at least two individuals, responsibility for the majority of housework, primary caregiver status, and urban residence. Behavioral variables (all yes/no) included chronic medication use, alcohol consumption (> 1 drink per week), smoking status (all respondents who did not select “I am a non-smoker”), low physical activity (< 1 time per week), and lived experience of sexual discrimination. Sexual behavior variables (all yes/no) included masturbation and partnered sexual activity within the past 12 months, experience of sexual trauma, and time spent in emotionally close relationships within the past 12 months. Operationalization and measurement details follow those described in Kronthaler et al. [46].

#### ICD-11 Screener for sexual problems and sexual dysfunction

SP and SD in men were assessed according to ICD-11 using the Screening tool for Sexual Problems in Men (SSP-M; [49] in German language. The assessment included eight questions, with four addressing the occurrence of SP within the past 6 months across the following domains: reduced desire, erectile dysfunction, delayed ejaculation, and early ejaculation. Responses were recorded on a 5-point Likert scale, ranging from not at all (1), episodically (2), sometimes (3), often (4), to always (5). SP was defined as the presence of any reported SP, regardless of frequency or concomitant sexual distress – yielding a binary SP variable (1 = presence of at least one SP; 0 = no SP). Upon SP indication, an additional item was presented to assess the level of sexual distress associated with SP. Participants were provided with the following response options to rate sexual distress: not at all (1), a bit (2), partly (3), severe (4), or very severe (5).

SD was defined as a SP in any of the four domains occurring at least episodically, accompanied by severe or very severe distress. Subsequently, SD was coded as a binary SD variable with (1) Yes (i.e., SP + severe or very severe sexual distress) and (0) No (i.e., no SP or SP but no significant concomitant sexual distress).

#### Sexual distress scale

In order to measure sexual distress, the Female Sexual Distress Scale-Desire/Arousal/Orgasm (FSDS-DAO) [50] was translated into German and the instructions were adapted for use in all genders (SDS-DAO). The translation to German language adhered to the linguistic validation guidance provided by the Mapi Group [51] and is subject to an additional publication [47]. Previous versions of the FSDS have been applied and validated in men, yielding psychometric properties comparable to those in women, as the items are formulated gender‑neutrally [52–54]. The FSDS-DAO consists of 15 items appraising the level of sexual distress that had occurred within the past 30 days. Response options are presented on a Likert scale ranging from never (0) to always [4], yielding a sum score between 0 and 60, with higher scores indicating higher sexual distress [50]. In the present sample, the SDS-DAO demonstrated excellent internal consistency (Cronbach’s α = 0.96).

### Statistical analysis

All statistical analyses were conducted using R (version 4.3.2), employing the UpSetR [60] and IsingFit [55] packages. Weighted descriptives (*M*, *SD*, frequencies) were calculated in addition to unweighted frequencies to ensure the representativeness of the sample in terms of age, gender, and federal state based on Microcensus 2014 [56]. Frequencies were provided for disjoint CHC and comorbid CHC subgroups. A comorbidity analysis was performed to visualize overlap among CHC subgroups. Descriptive prevalence estimates of SP, SD, and SDS-DAO were calculated and compared across the following groups: men with vs. without CHC, men with CHC with vs. without a comorbid mental health condition (MH + vs. CHC MH–), and each specific CHC subgroup individually. Odds ratios (ORs) with 95% confidence intervals (CIs) are reported for these group comparisons. To examine whether CHC was associated with SD or sexual distress, three regression analyses were conducted. Specifically, the associations between CHC status (model 1), MH status (model 2) and CHC subgroups (model 3; MH, CV, UR, IN, CA, PA, NE), coded as dichotomous variables (yes/no), and SD were analyzed using multivariable logistic regression with results reported as ORs with 95% CIs. To examine associations between CHC status and sexual distress (model 4), MH status (model 5), and CHC subgroups (model 6), three linear regression analyses were conducted, with results reported as regression coefficients (β) and corresponding 95% CI. All models included age, occurrence of 12-month engagement in sexual activity, and relationship status as covariates. Given the exploratory nature of the subgroup analyses, no correction for multiple testing was applied; overly conservative corrections risk obscuring potentially meaningful patterns that warrant follow-up investigation [57]. As such, p-values are reported to highlight potential associations and should be interpreted with caution.

In a next step, the interrelationships between CHC subgroups and SP, SD, individual domains of SP (i.e., sexual desire problems, erectile dysfunction, delayed ejaculation, early ejaculation problems), and individual domains of SD (i.e., hypoactive sexual desire dysfunction (HSDD), ED, male delayed ejaculation dysfunction (DE), and male early/premature ejaculation dysfunction (PE) were analyzed using network analysis for binary data as posited by previous research [58]. Accordingly, the Ising model was implemented with a regularized estimation through nodewise logistic regressions. To optimize selection, the OR rule and an EBIC hyperparameter (*γ* = 0.25) were employed. Interaction parameters (β) quantify the strength of associations between variables, while threshold parameters reflect the likelihood of variable occurrence within the sample. Four network models were displayed for total SP (Network A), total SD (Network B), SP domains (Network C), and SD domains (Network D). Missing responses to certain items were treated as missing data without imputation.

## Results

### Descriptive statistics

**Fig. 2:**
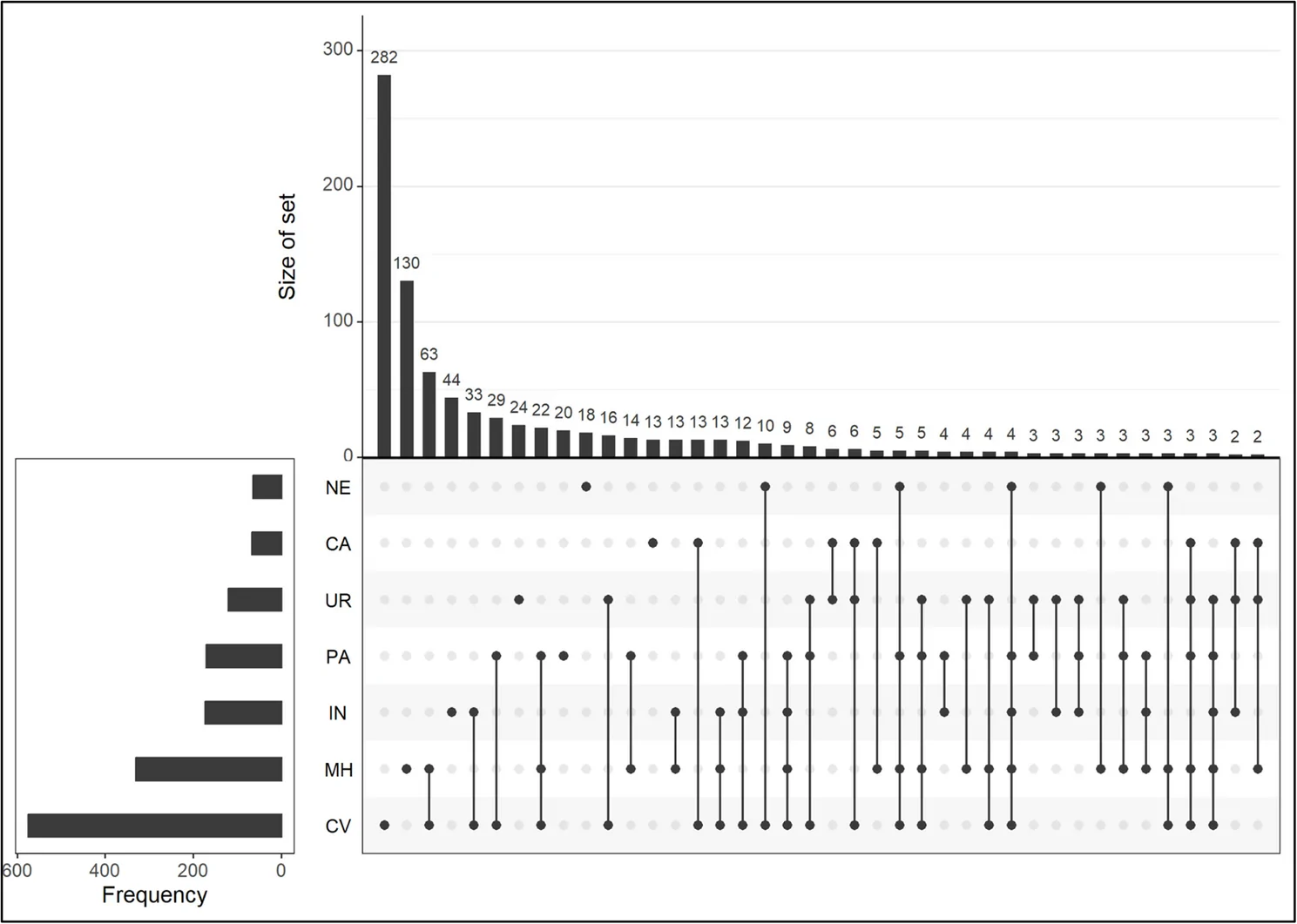
Comorbidity analysis of CHC subgroups (unweighted frequencies). Note. CHC: chronic health condition(s). CV: cardiovascular and metabolic CHC. MH: mental CHC. IN: infectious and inflammatory CHC. PA: pain-related CHC. UR: urological CHC. CA: cancer CHC. NE: neurological CHC. Set intersections are visualized in the upper bar plot, while the frequency of a CHC, irrespective of comorbidity, is visualized in the left bar plot

Out of *n =* 1742 (weighted *n* = 1768) participants, about half reported to be diagnosed with at least one CHC (*n* = 897; 50.8%, see Table 1). Men with CHC with comorbid mental health conditions (*n =* 331, 19.5%) had a mean age of *M* = 49.8 (*SD* = 14.9), while men with CHC without a comorbid mental health condition (*n =* 530, 31.2%) had a mean age of *M* = 58.2 (*SD* = 15.1; Table 1). Men with SD (*n =* 265, 16.6%, *M* = 42.9 years, *SD* = 14.8) were, on average, younger than men without SD (*n =* 1334, 83.4%, *M* = 51.5 years, *SD* = 17.1). Among all participants, the majority reported being in a relationship (61.2%). Multiple CHCs were present in *n =* 369 (20.9%) participants, with cardiovascular and mental health conditions being the most frequent comorbidity (Fig. 2). With a prevalence rate of 32.9%, cardiovascular and metabolic conditions formed the most common CHC subgroup, followed by mental health (21%), infection and inflammation (10.5%), and pain conditions (10.5%). All other CHC subgroups demonstrated prevalence rates below 10% (see Table 2).

**Table 1:** Characteristics of the study population.^a^

	No CHC	CHC		
		all	MH+	CHC MH-
N	870	897	331	530
Sociodemographics, n (%)				
Age (years), mean (SD)	44.0 (16.4)	55.0 (15.6)	49.8 (14.9)	58.2 (15.1)
Age (years) groups				
18–30	211 (24.3)	75 (8.4)	41 (12.1)	31 (5.9)
31–40	179 (20.6)	111 (12.4)	55 (16.3)	51 (9.8)
41–50	169 (19.4)	139 (15.5)	69 (20.6)	67 (12.9)
51–65	197 (22.6)	302 (33.6)	120 (35.7)	169 (32.4)
> 65	115 (13.2)	270 (30.1)	51 (15.3)	204 (39.1)
Education, > 12 years	510 (58.6)	389 (43.3)	132 (39.3)	241 (46.3)
Monthly netto income				
<€2500	430 (60.4)	558 (69.3)	248 (80.0)	292 (62.9)
€2500–5000	254 (35.6)	213 (26.5)	56 (18.0)	145 (31.3)
>€5000	28 (4.0)	33 (4.2)	6 (2.0)	27 (5.9)
Employed	549 (67.6)	357 (41.1)	126 (38.8)	218 (42.8)
Religious	435 (52.7)	482 (55.0)	182 (55.2)	276 (54.4)
Relationship	511 (59.7)	562 (62.7)	165 (49.0)	369 (70.9)
Sexual orientation				
Heterosexual	751 (92.4)	727 (85.4)	270 (84.1)	425 (86.4)
Homosexual	23 (2.9)	46 (5.4)	20 (6.1)	26 (5.3)
Bisexual	23 (2.8)	55 (6.5)	25 (7.9)	28 (5.7)
Migration background	127 (14.7)	102 (11.4)	44 (13.2)	51 (9.7)
Children, ≥ 1 in same household	201 (23.0)	168 (18.7)	71 (21.0)	87 (16.7)
Household size, ≥ 2	633 (72.8)	661 (73.7)	218 (64.9)	412 (79.0)
Majority of housework	198 (26.1)	262 (30.5)	115 (35.5)	140 (27.7)
Primary caregiver	21 (2.7)	46 (5.4)	17 (5.4)	28 (5.5)
Urban area	357 (41.0)	353 (39.4)	144 (42.9)	190 (36.5)
Behavioral risk factors, n (%)				
Medication for CHC	84 (11.0)	471 (54.2)	170 (52.5)	288 (56.3)
Alcohol consumption^c^	237 (30.8)	250 (28.8)	80 (24.6)	159 (31.1)
Smoking	353 (45.9)	416 (47.9)	173 (53.4)	227 (44.3)
Low physical activity^c^	342 (39.3)	289 (32.2)	106 (31.4)	172 (33.0)
Sexual discrimination	8 (1.1)	17 (2.0)	10 (3.0)	7 (1.4)
Sexual Behavior, n (%)				
Masturbation^b^	407 (53.0)	468 (53.8)	207 (63.8)	254 (49.7)
Partnered sexual activity^b^	244 (31.7)	197 (22.6)	85 (26.3)	106 (20.6)
Sexual trauma^b^	10 (1.3)	26 (3.0)	13 (3.9)	13 (2.6)
Spending time in relationships^b^	295 (38.8)	336 (39.2)	98 (30.3)	228 (45.0)

Note. *CHC *Chronic health condition(s),* MH+ *CHC with at least one comorbid mental health condition, *CHC MH*– CHC without any comorbid mental health condition

^a^weighted frequencies, group sizes are unweighted

^b^in the past 12 months

^c^< once per week

**Table:** 

	CV	MH	UR	IN	PA	CA	NE
Total *N*	1714	1603	1603	1603	1603	1603	1714
Comorbid CHC	564 (32.9)	336 (21.0)	120 (7.5)	169 (10.5)	169 (10.5)	65 (4.1)	65 (3.8)
Disjoint CHC	275 (16.0)	140 (8.7)	25 (1.6)	43 (2.7)	21 (1.3)	13 (0.8)	17 (1.0)

Note. *CHC *Chronic health condition(s), *CV *Cardiovascular and metabolic CHC, *MH *Mental CHC, *UR *Urological CHC, *IN *Infectious and inflammatory CHC, *PA *Pain-related CHC, *CA *Cancer CHC, *NE *Neurological CHC

In the last 12 months, 26.9% of men indicated to have engaged in sexual activity with another person, while 53% indicated to have engaged in masturbation or sexual activity with themselves. Sexual activity rates were distinct between different CHC groups. Men with inflammatory conditions (38.8% sexually active), mental health conditions (31.7%), and no CHC (31.7%) had engaged in sexual activity most frequently, while men with cancer (10.0%) and neurological conditions (4.5%) showed the lowest engagement rates.

### Prevalence of SP and SD symptoms in distinct CHC subgroups

**Fig. 3:**
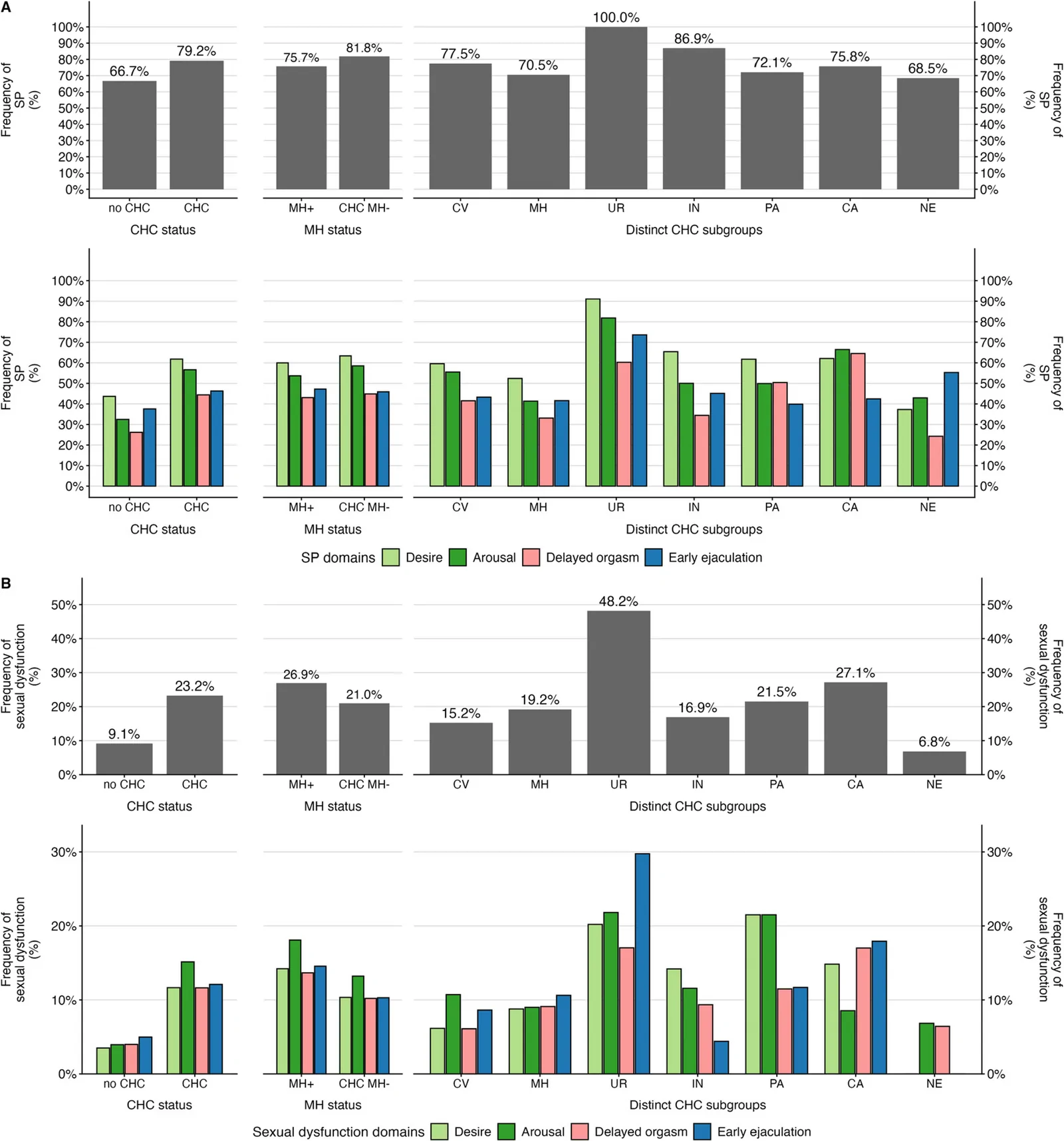
Prevalence of SP and SP domains by CHC status and disjoint CHC subgroups. Note. SP, sexual problem. CHC: chronic health condition(s). MH+: CHC with at least one comorbid mental health condition. CHC MH–: CHC without any comorbid mental health condition. CV: cardiovascular and metabolic CHC. MH: mental CHC. UR: urological CHC. IN: infectious and inflammatory CHC. PA: pain-related CHC. CA: cancer CHC. NE: neurological CHC

Men with CHC reported higher 6-month prevalence rates of SP compared to men without CHC (79.2% vs. 66.7%, OR 1.90, 95% CI 1.52–2.37), with a similar pattern in sexually active men (84.0% vs. 73.0%, OR 1.95, 95% CI 1.21–3.19; see Table 3). In both groups, reduced sexual desire was the most frequently reported SP. Regarding CHC subgroups, SP was most prevalent among men with urological conditions (100%), followed by inflammatory (86.9%), cardiovascular (77.5%), cancer (75.8%), pain (72.1%), mental health (70.5%), and neurological conditions (68.5%; see Fig. 3 and Supplement Table S1).

In line with SP, SD was also more prevalent in the CHC group among all men (23.2% vs. 9.1%, OR 3.00, 95% CI 2.24–4.07) and sexually active men (22.0% vs. 10.9%, OR 2.30, 95% CI 1.35–3.99), with ED being the most affected SD domain across both samples (all men: 15.1% vs. 3.9%, OR 4.34, 95% CI 2.92–6.64; sexually active men: 14.1% vs. 3.7%, OR 4.21, 95% CI 1.99–9.83). For detailed prevalence rates, ORs, and 95% CIs for SP and SD among all men and sexually active men by CHC compared to the non-CHC group, see Table 3. For frequency statistics of the single SP and SD domains by CHC subgroup, see Fig. 3 and Supplement Table S1.

**Table 3:** *SP and SD prevalence among all men and sexually active men by CHC status in n (%).*

	No CHC		CHC		OR (95% CI)	
	All	Sexually active	All	Sexually active	All	Sexually active
*n*	775	238	868	196	-	-
SP, any	517 (66.7)	174 (73.0)	687 (79.2)	165 (84.0)	1.90 (1.52–2.37)	1.95 (1.21–3.19)
Desire problems	344 (43.7)	106 (44.0)	538 (61.8)	124 (63.4)	2.09 (1.71–2.55)	2.21 (1.50–3.27)
Erectile problems	253 (32.5)	78 (32.8)	490 (56.6)	108 (55.4)	2.72 (2.22–3.33)	2.55 (1.72–3.79)
Delayed ejaculation problems	203 (26.2)	59 (25.0)	383 (44.4)	78 (39.9)	2.25 (1.83–2.78)	2.00 (1.32–3.03
Early ejaculation problems	290 (37.6)	105 (44.0)	397 (46.3)	88 (45.2)	1.43 (1.17–1.75)	1.05 (0.71–1.54)
*n*	753	235	847	194	-	-
SD, any	69 (9.1)	26 (10.9)	197 (23.2)	43 (22.0)	3.0 (2.24–4.07)	2.3 (1.35–3.99)
Hypoactive sexual desire dysfunction	27 (3.5)	10 (4.3)	101 (11.7)	15 (7.7)	3.63 (2.38–5.72)	1.84 (0.81–4.32)
Male Erectile dysfunction	31 (3.9)	9 (3.7)	130 (15.1)	27 (14.1)	4.34 (2.92–6.64)	4.21 (1.99–9.83)
Male delayed Ejaculation dysfunction	31 (4.0)	11 (4.5)	100 (11.6)	20 (10.0)	3.17 (2.11–4.90)	2.34 (1.09–5.27)
Male early Ejaculation dysfunction	38 (5.0)	11(4.4)	103 (12.1)	21 (11.0)	2.63 (1.80–3.91)	2.67 (1.26-6.00)

Note. Sexually active: participants reporting partnered sexual activity within the past 12 months

*CHC* Chronic health condition(s), *SD* Sexual dysfunction

### Prevalence of Sexual Distress in distinct CHC subgroups

**Fig. 4:**
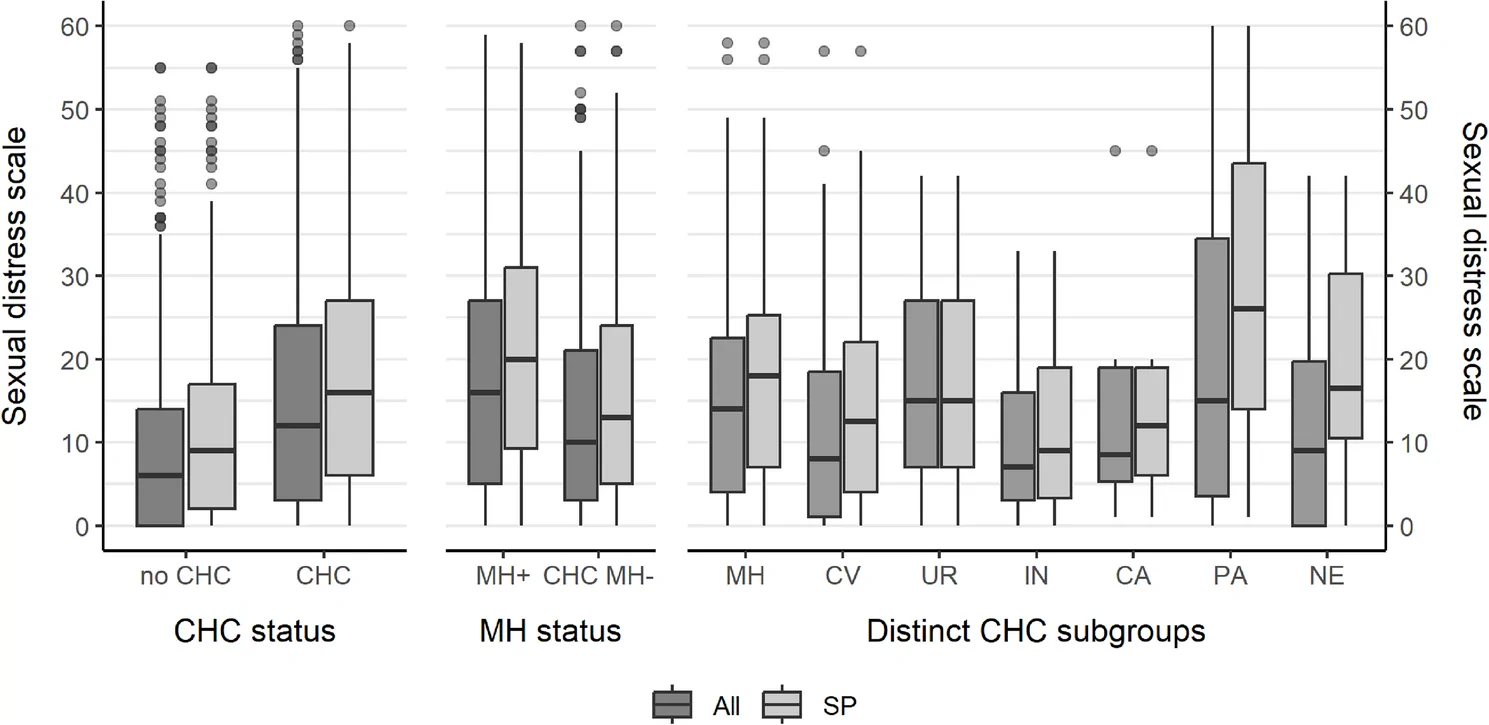
Sexual Distress (SDS-DAO score) by CHC status and CHC subgroups. *Note. SDS-DAO score measured on a scale from 0 to 60, with higher scores indicating higher distress. CHC: chronic health condition(s). MH+: CHC with at least one comorbid mental health condition. CHC MH–: CHC without any comorbid mental health condition. CV: cardiovascular and metabolic CHC. MH: mental CHC. UR: urological CHC. IN: infectious and inflammatory CHC. PA: pain-related CHC. CA: cancer CHC. NE: neurological CHC

Descriptively, mean sexual distress was higher in men with CHC (*M* = 14.8 (*SD* = 13.5)), compared to men without CHC (*M* = 9.3 (*SD* = 11.1)). In men with SP, sexual distress was descriptively higher with *M* = 17.5 (*SD* = 13.4) in men with CHC and *M* = 12.0 (*SD* = 11.6) in men without CHC. Further, in men with SP, the pain subgroup reported the highest sexual distress scores with *M* = 20.8 (*SD* = 19.5), followed by urological (*M* = 17.1 (*SD* = 11.5)), mental health (*M* = 15.5 (*SD* = 13.1)), neurological (*M* = 13.0 (*SD* = 13.9)), cancer (*M* = 12.7 (*SD* = 12.4)), cardiovascular (*M* = 11.5 (*SD* = 11.6)), and inflammatory (*M* = 10.8 (*SD* = 9.2)) subgroups, see Fig. 4. For Sexual Distress (SDS-DAO) scores by CHC, see Supplement Table S2.

### Association of CHC status, MH status, and comorbid CHC subgroups with SD

Three separate multivariable logistic regression models examined associations between CHC status (model 1), MH status (model 2), and CHC subgroups (model 3) and SD, adjusting for age, 12-month partnered sexual activity, and relationship status (see Table 4).

**Table 4:** Results of multivariable logistic regression models for SD, with separate models including CHC status, MH status, and comorbid CHC subgroups. Estimates include OR, 95% CIs, and associated p-values

	Model 1 (*n* = 1578)		Model 2 (*n* = 822)		Model 3 (*n* = 1578)	
	OR (95% CI)	*P* value	OR (95% CI)	*P* value	OR (95% CI)	*P* value
CHC [yes]	3.5 (2.6–4.9)	< 0.001				
Age	1.00 (1.0–1.0)	0.001	1.00 (1.0–1.0)	0.001	1.0 (1.0–1.0)	< 0.001
Sexual activity	1.00 (0.7–1.3)	0.765	0.8 (0.5–1.1)	0.171	1.0 (0.7–1.3)	0.804
Relationship	1.00 (0.7–1.3)	0.873	0.9 (0.7–1.3)	0.711	1.0 (0.8–1.4)	. 914
MH+/CHC MH- [MH]			1.2 (0.8–1.7)	0.332		
CV+					**2.1 (1.5–2.9)**	**< 0.001**
MH+					**2.0 (1.4–2.9)**	**< 0.001**
UR+					**3.9 (2.5–6.2)**	**< 0.001**
IN+					1.1 (0.7–1.7)	0.786
CA+					**2.5 (1.3–4.6)**	**0.004**
PA+					1.0 (0.6–1.5)	0.874
NE+					1.1 (0.5–2.1)	0.812

Note. *OR *Odds ratio, *CI *Confidence interval, *CHC *Chronic health condition(s), *MH+ *CHC with at least one comorbid mental health condition, *CHC MH– *CHC Without any comorbid mental health condition, *CV+ *Comorbid cardiovascular and metabolic CHC, *MH+ *Comorbid mental health CHC, *UR+ *Comorbid urological CHC, *IN+ *Comorbid infectious and inflammatory CHC, *CA+ *Comorbid cancer-related CHC, *PA+ *Comorbid pain-related CHC, *NE+ *Comorbid neurological CHC

^a^Partnered sexual activity in the last 12 months

Model 1 showed that men with CHC were more likely to report SD compared to men without CHC (OR 3.5, 95% CI 2.6–4.9, *p* < .001). Model 2 indicated no significant association between MH status and SD (OR 1.2, 95% CI 0.8–1.7, *p* = .332). Model 3 demonstrated significant positive associations with SD for urological (OR 3.9, 95% CI 2.5–6.2), cancer (OR 2.5, 95% CI 1.3–4.6), cardiovascular (OR 2.1, 95% CI 1.5–2.9), and mental health conditions (OR 2.0, 95% CI 1.4–2.9), while infectious/inflammatory (OR 1.1), pain (OR 1.0), and neurological conditions (OR 1.1) were not significantly associated with SD (all *p* > .05; see Table 4).

### Association of CHC status, MH status, and comorbid CHC subgroups with sexual distress

Three separate multivariable linear regression models examined associations between CHC status (model 4), MH status (model 5), and CHC subgroups (model 6) and sexual distress, adjusting for age, 12-month partnered sexual activity, and relationship status (see Table 5).

**Table 5:** Results of linear regression models for sexual distress, with separate models including CHC status, MH Status and comorbid CHC subgroups. Regression coefficients (β), CIs, and p-values are reported

	Model 4 (*n* = 1519)		Model 5 (*n* = 782)		Model 6 (*n* = 1519)	
	β – coefficients (95% CI)	*P* value	β – coefficients (95% CI)	*P* value	β – coefficients (95% CI)	*P* value
CHC [yes]	6.3 (5.0–7.6)	< 0.001				
Age	-0.1 (-0.1 – -0.0)	0.001	-0.1 (-0.2 – -0.0)	0.003	-0.1 (-0.1 – -0.0)	0.001
Sexual activity^a^	-1.5 (-3.0 – -0.1)	0.034	-2.5 (-4.7 – -0.2)	0.031	-1.6 (-3.0 – -0.2)	0.027
Relationship	-0.7 (-2.0–0.7)	0.333	-1.3 (-3.3–0.8)	0.224	-0.4 (-1.7–0.9)	0.574
MH+/CHC MH- [MH]			3.4 (1.3–5.4)	0.001		
CV+					3.3 (1.9–4.8)	**< 0.001**
MH+					5.8 (4.1–7.4)	**< 0.001**
UR+					8.5 (6.0–11.0)	**< 0.001**
IN+					0.8 (-1.4–2.9)	0.482
CA+					4.1 (0.9–7.3)	**0.013**
PA+					1.6 (-0.6–3.8)	0.157
NE+					1.9 (-1.3–5.1)	0.241

Note. *CI *Confidence interval, *CHC *Chronic health condition(s), *MH+ *CHC with at least one comorbid mental health condition, *CHC MH– *CHC without any comorbid mental health condition, *CV+ *Comorbid cardiovascular and metabolic CHC, *MH+ *Comorbid mental health CHC, *UR+ *Comorbid urological CHC, *IN+ *Comorbid infectious and inflammatory CHC, *CA+ *Comorbid cancer-related CHC, *PA+ *Comorbid pain-related CHC, *NE+ *Comorbid neurological CHC

^a^Partnered sexual activity in the last 12 months

Model 4 showed that men with CHC reported significantly higher sexual distress compared to men without CHC (β = 6.3, 95% CI 5.0–7.6, *p* < .001). Model 5 indicated that comorbid mental health conditions were significantly associated with higher sexual distress (β = 3.4, 95% CI 1.3–5.4, *p* = .001). Model 6 demonstrated significant positive associations with sexual distress for urological (β = 8.5, 95% CI 6.0–11.0), mental health (β = 5.8, 95% CI 4.1–7.4), cancer (β = 4.1, 95% CI 0.9–7.3, *p* = .013), and cardiovascular conditions (β = 3.3, 95% CI 1.9–4.8), while infectious/inflammatory (β = 0.8), pain (β = 1.6), and neurological conditions (β = 1.9) were not significantly associated with sexual distress (all *p* > .05; see Table 5).

A post-hoc subgroup analysis revealed that the association between CHC and SD was lower in men in a relationship (OR 2.68, 95% CI 1.82–4.00, *p* = .001) compared to single men (OR 5.34, 95% CI 3.17–9.34, *p* < .001; see S3 in Supplement).

### Network analyses

**Fig. 5:**
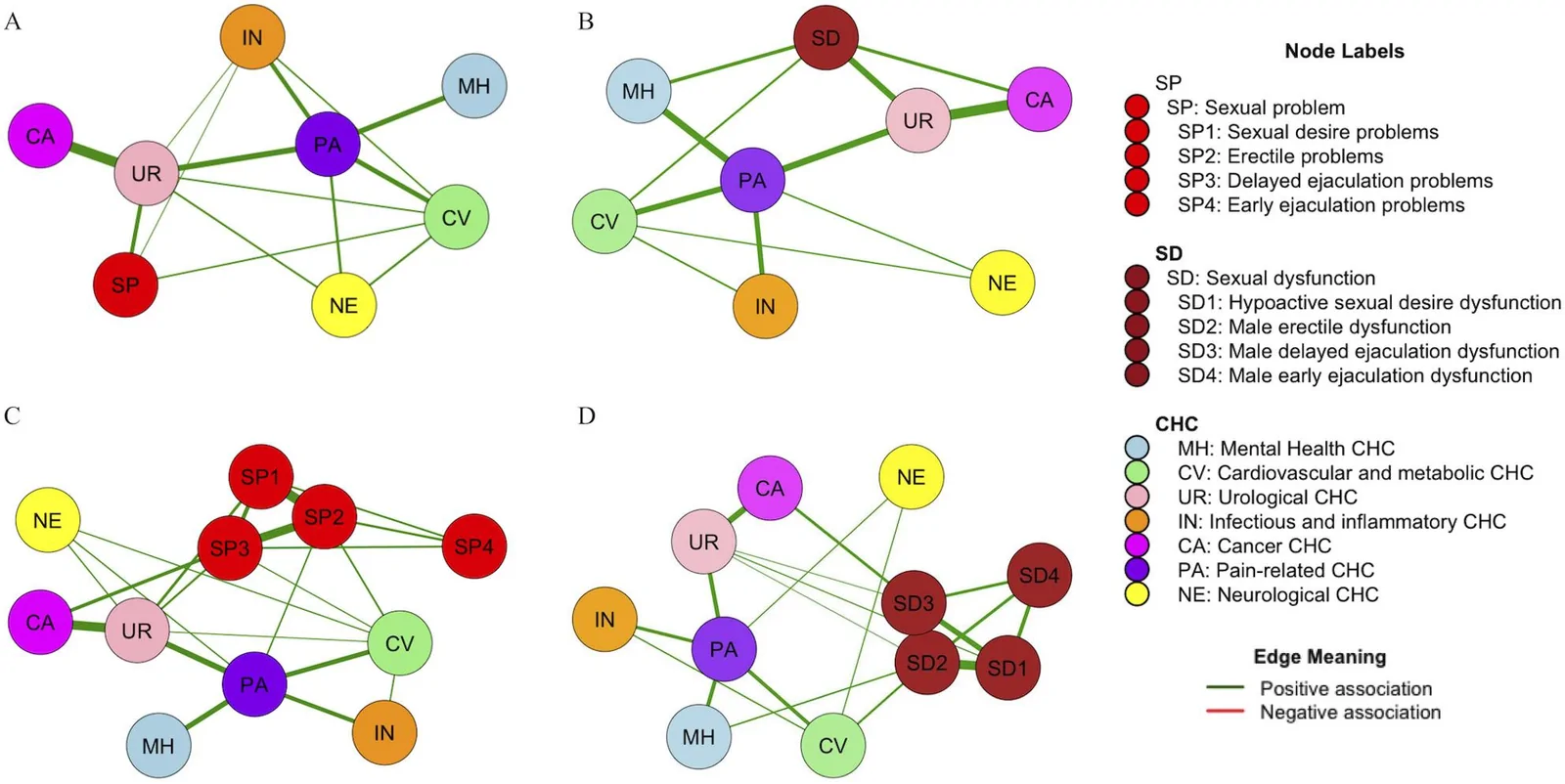
Network Models of Associations between SP, SD, and CHC conditions. *Note. Network analyses of CHC subgroups and total SP (A), total SD (B), individual domains of SP (C), and individual domains of SD (D)

The network analyses illustrate the comorbidity patterns of CHC subgroups with overall SP and SD, and the SP and SD subdomains are visually presented in Fig. 5. Using a hyperparameter of 0.25, notable associations emerged, including a link between SP (network A) and urological CHC (β_SPUR_ = 0.97) as well as between SP and cardiovascular and metabolic CHC (β_SPCV_ = 0.31).

The SP domains (network C) demonstrated strong intercorrelations, with the most pronounced relationships observed between desire and erectile problems **(**β_SP1SP2_ = 2.06) and erectile problems and delayed ejaculation (β_SP2SP3_ = 1.85). Specific associations with the SP domains included desire and urological conditions (β_SP1UR_ = 0.72), erectile problems and cardiovascular conditions (β_SP2CV_ = 0.41), delayed ejaculation and cancer (β_SP3CA_ = 0.70), and delayed ejaculation and urological conditions (β_SP3UR_ = 0.45).

Similarly, SD (network B) exhibited strong associations with urological conditions CHC (β_SDUR_ = 0.92), moderate associations with mental health CHC (β_SDMH_ = 0.50), cancer (β_SDCA_ = 0.47), and cardiovascular conditions (β_SDCV_ = 0.32).

As with SP, the SD domains also showed strong intercorrelations (network D), particularly between hypoactive sexual desire dysfunction (HSDD) and ED **(**β_SD1SD2_ = 2.60) and ED and male delayed ejaculation dysfunction (DE) (β_SD2SD3_ = 3.01). Moderate to strong associations with the SD domains were found for HSDD and urological CHC (β_SD1UR_ = 0.48), for ED with CV (β_SD2CV_ = 0.69), and MH (β_SD2MH_ = 0.41). For DE with cancer (β_SD3CA_ = 0.77).

For further details on the interaction parameter β, refer to Tables S4–S7 in the Supplement. Utilizing a spring layout, pain-related CHCs were centrally positioned across all networks and exhibited high regression coefficients with various conditions, particularly urological, cardiovascular, and mental health CHCs. Notably, cardiovascular CHCs had the highest threshold in networks B and C and SP and SP4 in networks A and C, respectively, highlighting their high probability of presence (Table S8, in the Supplement).

## Discussion

The present study aimed to compare the prevalence of SP, SD, and sexual distress among men with and without CHC, with and without comorbid mental health conditions, and different CHC subgroups. Further, associations between SD and CHC subgroups were modeled using regression and network analysis. The CHC frequency rates demonstrated that cardiovascular and metabolic conditions and mental health conditions were the most prevalent CHC subgroups. The 6-month prevalence rate of SP and SD was higher in men with CHC, compared to men without CHC; with 79.2% of men reporting SP (OR 1.90, 95% CI 1.52–2.37) and 23.2% reporting SD (OR 3.00, 95% CI 2.24–4.07). Erectile function was most frequently affected, with 15.1% of men with CHC experiencing ED. Sexual distress was highest in CHC subgroups with pain, urological, and mental health conditions. Comorbid mental health conditions most frequently co-occurred in men with cardiovascular conditions. Prevalence estimates were based on disjoint subgroups to ensure comparability across studies. Comorbid CHC subgroups were used for the regression analyses to examine the independent association of each CHC category with SD and sexual distress while accounting for the presence of other conditions. Overall, the regression and network models showed CHC (vs. no CHC) to be strongly associated with increased SD prevalence in men - with substantial variability across CHC subgroups. Specifically, urological, cardiovascular, and mental health conditions and cancer were consistently identified across both analytical approaches as being associated with higher SD prevalence and sexual distress. The presence of a comorbid mental health did not explain additional variance in SD. However, a comorbid mental health condition was significantly associated with higher sexual distress.

### CHC and sexual dysfunction and distress

The observed associations between CHC and SD corroborate previous findings, suggesting that sexual health is strongly related to overall health. Among the CHC subgroups, men with urological disorders reported the highest rates of SP and SD. Consistent with the regression analyses, the network analyses identified urological conditions as the CHC subgroup most strongly associated with SD. Furthermore, HSDD emerged as the SD domain most strongly connected to urological conditions in the network analyses, suggesting that reduced sexual desire may represent a particularly salient aspect of sexual dysfunction in men with urological conditions. Given the direct involvement of urological health in sexual function, this finding aligns with previous research highlighting the adverse effects of conditions such as pelvic floor dysfunction, incontinence, and infertility on sexual desire, erectile function, and ejaculatory control [59–61]. However, evidence suggests that male infertility (present in 14.6% of mn with urological conditions) is itself associated with an increased prevalence of SD [62]. Therefore, the overlap between infertility-related SD and other urological conditions may have contributed to the elevated prevalence estimates and associations observed in this subgroup, which should be interpreted with caution, as they may partly reflect infertility-related SD rather than urological conditions more broadly. The underlying pathophysiological mechanisms likely involve disruptions in pelvic innervation, vascular insufficiency, and endocrine alterations. Results are in line with other studies finding that 70% of men in treatment for lower urinary tract infections experience erectile and ejaculatory dysfunction [58–60].

Similarly, men with cardiovascular and metabolic conditions demonstrated markedly increased SD prevalence, particularly ED. This pattern was further supported by both the regression and network analyses, in which cardiovascular conditions showed moderate association with SD. Notably, ED emerged as the SD domain most strongly associated with cardiovascular conditions in the network analysis, highlighting the particularly close relationship between cardiovascular health and erectile functioning. This is consistent with established pathophysiological links between vascular dysfunction and endothelial impairment, which reduce nitric oxide bioavailability and thereby compromise erectile function [59, 60]. Given that ED is often an early indicator of systemic vascular disease, these findings emphasize the importance of routine sexual health assessments as part of cardiovascular risk stratification [60, 61].

Cancer diagnosis and treatment were also related to higher odds of reporting SD, as reflected by the elevated odds ratios observed in the regression analyses. This finding was corroborated by the network analysis, in which cancer showed moderate associations with SD overall. Furthermore, cancer was particularly associated with DE, representing one of the strongest CHC-specific connections observed at the SD domain level. Therefore, present results correspond to the wide range of literature showing the adverse effects of cancer treatment on male sexual function [63]. Forms of pelvic cancer such as prostate or testicular cancer may involve treatment types and surgical complications directly affecting the central pelvic area integral to the maintenance of sexual function [64]. Further, cancer treatments may affect erectile function, testosterone production, and ejaculatory function via neurological changes [64]. While still underdiagnosed and underreported, SD is widely reported across cancer diagnoses, age groups, and treatment types [63, 64]. The present results are in line with a wide array of research showing that cancer diagnoses, treatment, and consequential bodily changes may elicit substantial (sexual) distress [63, 65].

### Mental Health and Sexual Dysfunction and Distress

Mental health conditions exhibited heightened odds of SD, and a significant relationship with sexual distress. Further, MH status (i.e., CHC with comorbid mental health condition) was associated with increased sexual distress. An analysis of the distribution of mental health conditions in the MH+ group revealed high prevalence rates of depression (73.7%) and anxiety disorders (40.5%; see Supplement Table S9). The present findings are consistent with previously postulated bidirectional associations between mental health disorders and SD [39] and underscore the independent contribution of (comorbid) MH conditions to SD. The network analyses further supported these findings, identifying mental health conditions as the CHC subgroup most strongly associated with SD after urological conditions. At the domain level, mental health conditions showed their strongest association with ED, suggesting that erectile difficulties may represent an important manifestation of the relationship between mental health and sexual functioning. This may be due to the direct somatic effects of mental health disorders such as depression and their medications can have on sexual functioning in general and erectile functioning in particular, to psychological symptoms such as anhedonic tendencies in depression or hypervigilance in anxiety, or to the common pathophysiological processes that are involved in depression, erectile dysfunction, and cardiovascular conditions [66–68]. Further, pain conditions seemed strongly related to mental health conditions, which are suggested to have a bidirectional relationship that emanates from shared neural mechanisms and effects on lifestyle choices and quality of life [69]. Although pain was not directly associated with SD or sexual distress, the network models depicted strong relationships between mental health and pain conditions and ED. A supplementary post-hoc analysis confirmed that pain-related CHC did not explain significant additional variance in SD over and above the other CHC subgroups (F = 0.03, *p* = .87; see Supplement Table S10). Therefore, while pain conditions such as chronic pain, chronic pelvic pain, and bladder pain syndrome are centrally connected to other CHC, such as mental health, urological, and cardiovascular conditions, no direct association with SD diagnosis or sexual distress was shown. This may be due to pain being an accompanying factor of various CHC, rather than an isolated condition. Therefore, pain conditions may be strongly related to individual CHC while not adding additional explained variance in SD and sexual distress. For example, literature finds depression prevalence rates of up to 60% across chronic pain groups [69] Given the strong connections between pain and other CHC, such as cardiovascular, inflammatory, urological, and mental health, it is reasonable to infer that in other CHC subgroups, chronic pain may be associated with both MH conditions and SD, and therefore comprises a common underlying factor that is relevant to consider in the treatment of CHC and SD. Germane to this, pain-related CHC emerged as a particularly salient correlate of sexual distress, aligning with prior research linking chronic pain conditions to impairments in sexual function due to a combination of physiological, psychological, and relational factors [70].

Lastly, in men with CHC, being in a relationship was associated with lower odds of reporting SD. This corresponds with research showing that being in a relationship is positively associated to health-related quality of life in men [71]. However, this may be moderated by relationship quality, as dysfunctional or conflictual relationships have been found to contribute to SD in men [72].

### Implications for clinical practice and public health

The findings have critical implications for clinical practice. Despite the high prevalence of SD in men with CHC, sexual health remains under prioritized in routine medical care, often due to time constraints, stigma, and a lack of provider training [14, 21]. Given the strong associations between CHC and SD, systematic sexual health screenings should be integrated into primary care and specialty settings, particularly for patients with cardiovascular, urologic, and mental health disorders and post-cancer treatment. Furthermore, the sexual distress that is elicited by aforementioned conditions, as well as by chronic pain, highlights the need for interdisciplinary, patient-centered interventions that incorporate psychological support alongside medical treatment. In light of the established adverse effects of mental health conditions on sexual functioning, the heightened risk of developing comorbid mental health conditions when affected by CHC should be targeted in specialized care such as cardiology, urology, and oncology.

From a public health perspective, these findings reinforce the necessity of targeted sexual health education and intervention programs tailored to individuals with CHC. Increased awareness among healthcare providers and patients regarding the association of CHC with sexual function could facilitate more proactive management and improve patient outcomes. Partner-based approaches are of particular importance from a public health perspective, as relationship status was associated with lower odds of SD among men with CHC. The finding that being in a relationship appears to be associated with lower probability of SD for men with CHC underscores the value of incorporating relationship dynamics into treatment approaches. The integration of partner-inclusive interventions into standard care pathways could enhance treatment outcomes and quality of life for affected individuals. This approach aligns with the growing recognition that SD impacts not only the individual but also their intimate relationships.

### Strengths, limitations, and future directions

This study represents a large-scale assessment of the association between CHC and SD in men, leveraging a representative sample and validated diagnostic criteria that include sexual distress as relevant criterion. The use of network analysis further allowed for nuanced exploration of CHC comorbidities and their interaction with SD domains, providing novel insights into the complexity of these relationships.

However, several limitations should be noted. First, the sample was only representative of the selected variables, namely gender, age, and federal state. Therefore, other variables may differ from the general population, causing deviations in the prevalence of SD. Second, the cross-sectional design precludes causal inferences, necessitating longitudinal studies to clarify temporal relationships between CHC onset and SD development. In addition, the survey-based, cross-sectional design did not allow us to disentangle the relative contributions of CHC, their treatment, comorbid mental health conditions, and other etiological factors to the reported SD. Consistent with the ICD-11 framework, which conceptualizes sexual dysfunction as multifactorial and records medical, substance- or medication-related, psychological, and relational contributors through non-mutually-exclusive aetiological qualifiers, our cross-sectional, self-report data did not permit assignment of these aetiological contributors at the individual level. Third, although the study used validated self-report measures, relying on subjective reporting may introduce recall and response biases. In addition, CHC diagnoses were assessed exclusively by self-report and could not be clinically verified, potentially resulting in diagnostic misclassification. Such misclassification may have affected the reported prevalence estimates and the strength of the observed associations. Objective clinical assessments, including hormonal profiling and vascular testing, could not be applied in this study and may be useful for enhancing diagnostic precision in future research. Fourth, the network analyses were exploratory and intended to complement the main prevalence and regression analyses. As no formal network stability, edge-weight accuracy, or robustness analyses were conducted, the observed network structure should be interpreted with caution and requires further validation, including formal stability testing, in future studies. Fifth, several subgroup prevalence estimates, particularly for cancer, neurological, and urological conditions, were based on relatively small sample sizes and should therefore be interpreted cautiously. Small subgroup sizes yield wider confidence intervals and less stable estimates. Sixth, men with CHC were, on average, 11 years older than men without CHC. Despite having included age as a covariate in the regression analyses, this substantial age difference between men with and without CHC may have influenced the observed associations, and residual confounding by age cannot be excluded. Lastly, given the exclusively German-speaking sample, the exclusion of non-binary individuals, and the absence of intersectional analyses by sexual orientation, future studies should examine SD disparities among various demographic, gender, and sexual identity groups to enhance generalizability.

## Conclusion

In conclusion, the present findings support the postulate that CHC are substantially associated with a higher prevalence and higher odds of SD in men and highlight the important and positive association between urological and mental health conditions and male SD, alongside the well-established links of cardiovascular conditions and cancer to male SD. The strong associations with sexual distress further underscore the profound psychosocial impact of SD, necessitating a paradigm shift in healthcare toward more comprehensive, patient-centered approaches to sexual health. By prioritizing sexual health assessments and integrating multidisciplinary interventions that include partner-based approaches, healthcare systems may better address the unmet needs of individuals with CHC.

## Supplementary Information

Supplementary Material 1.

## Data Availability

The datasets analyzed during this study are not publicly available due to restrictions imposed by the ethics approval.

## Abbreviations

AIDS: Acquired immunodeficiency syndrome
CA: Cancer chronic health conditions
CHC: Chronic health condition
CHC MH−: Chronic health conditions excluding mental health conditions
CHC MH+: Chronic health conditions with comorbid mental health condition
CI: Confidence interval
CV: Cardiovascular and metabolic chronic health conditions
DE: Delayed ejaculation
ED: Erectile dysfunction
FSDS-DAO: Female Sexual Distress Scale-Desire/Arousal/Orgasm
HIV: Human immunodeficiency virus
HSDD: Hypoactive sexual desire disorder
ICD-11: International Classification of Diseases, 11th Revision
IN: Infectious and inflammatory chronic health conditions
MH: Mental health–related chronic health conditions
NE: Neurological chronic health conditions
OR: Odds ratio
PA: Pain-related chronic health conditions
PE: Premature ejaculation
PSE: Patient and stakeholder engagement
SD: Ptandard deviation
SDS-DAO: Sexual Distress Scale-Desire/Arousal/Orgasm
SP: Problems in sexual function
STROBE: Strengthening the Reporting of Observational Studies in Epidemiology

## References

[1] Organization WH. Defining sexual health report of a technical consultation on sexual health 28–31 January 2002, Geneva. Sexual health document series. World Health Organization; 2006. Available from: http://www.who.int/reproductive-health/publications/sexual_health/defining_sexual_health.pdf

[2] Briken P, Matthiesen S, Pietras L, Wiessner C, Klein V, Reed GM, et al. Estimating the prevalence of sexual dysfunction using the new ICD-11 guidelines: Results of the first representative, population-based German health and sexuality survey (GeSiD). Dtsch Arztebl Int. 2020;117(39):653. PubMed PMID: 33357346. 10.3238/arztebl.2020.0653 PMC7829447 33357346

[3] Gebeyehu NA, Gesese MM, Tegegne KD, Kebede YS, Kassie GA, Mengstie MA, et al. Global prevalence of sexual dysfunction among diabetic patients from 2008 to 2022: systematic review and meta-analysis. Metabol Open. 2023;18:100247. 10.1016/j.metop.2023.100247 PMC10267599 37323562

[4] Herder T, Spoelstra SK, Peters AWM, Knegtering H. Sexual dysfunction related to psychiatric disorders: a systematic review. J Sex Med. 2023;20(7):965–76. 10.1093/jsxmed/qdad074 37279603

[5] Beier KM, Loewit KK. Sexual medicine in clinical practice. Springer Science & Business Media; 2012.

[6] Flynn KE, Lin L, Bruner DW, Cyranowski JM, Hahn EA, Jeffery DD, et al. Sexual Satisfaction and the Importance of Sexual Health to Quality of Life Throughout the Life Course of U.S. Adults. J Sex Med. 2016;13(11):1642–50. .JSXM.2016.08.011 PubMed PMID: 27671968. 10.1016/j.jsxm.2016.08.011 PMC5075511 27671968

[7] ICD-11. Available from: https://icd.who.int/en/.[cited 2026 Jun 26].

[8] Briken P, Matthiesen S, Pietras L, Wiessner C, Klein V, Reed GM, et al. Estimating the Prevalence of Sexual Dysfunction Using the New ICD-11 Guidelines. Dtsch Arztebl Int. 2020. 10.3238/arztebl.2020.0653. PMC7829447 33357346

[9] Frikha F. Sexual Function and Dysfunction among Patients with Systemic and Auto-Immune Diseases. Ann Clin Med Case Rep. 2021;5(11):1–9.

[10] Puth MT, Weckbecker K, Schmid M, Münster E. Prevalence of multimorbidity in Germany: impact of age and educational level in a cross-sectional study on 19,294 adults. BMC Public Health. 2017;17:1–7. 10.1186/s12889-017-4833-3 PMC5648462 29047341

[11] Basson R, Rees P, Wang R, Montejo AL, Incrocci L. Sexual function in chronic illness. J Sex Med. 2010;7(1Part2):374–88. 10.1111/j.1743-6109.2009.01621.x 20092445

[12] Iovino P, Vellone E, Cedrone N, Riegel B. A middle-range theory of social isolation in chronic illness. Int J Environ Res Public Health. 2023;20(6):4940. 10.3390/ijerph20064940 PMC10049704 36981849

[13] Karsten MM, Roehle R, Albers S, Pross T, Hage AM, Weiler K, et al. Corrigendum to ‘Real-world reference scores for EORTC QLQ-C30 and EORTC QLQ-BR23 in early breast cancer patients [European Journal of Cancer 163 (2022) 128–139]. Eur J Cancer. 2022;174:325–7. 10.1016/j.ejca.2022.06.052 35970736

[14] Makovski TT, Schmitz S, Zeegers MP, Stranges S, van den Akker M. Multimorbidity and quality of life: systematic literature review and meta-analysis. Ageing Res Rev. 2019;53:100903. 10.1016/j.arr.2019.04.005 31048032

[15] Petitte T, Mallow J, Barnes E, Petrone A, Barr T, Theeke L. A systematic review of loneliness and common chronic physical conditions in adults. Open Psychol J. 2015;8(Suppl 2):113. PubMed PMID: 26550060. 10.2174/1874350101508010113 PMC4636039 26550060

[16] Goldstein I, Kim NN, Clayton AH, DeRogatis LR, Giraldi A, Parish SJ et al. Hypoactive sexual desire disorder: International Society for the Study of Women’s Sexual Health (ISSWSH) expert consensus panel review. In: Mayo clinic proceedings. Elsevier; 2017. pp. 114–28. 10.1016/j.mayocp.2016.09.018 27916394

[17] Goldstein I, Komisaruk BR, Pukall CF, Kim NN, Goldstein AT, Goldstein SW, et al. International Society for the Study of Women’s Sexual Health (ISSWSH) review of epidemiology and pathophysiology, and a consensus nomenclature and process of care for the management of persistent genital arousal disorder/genito-pelvic dysesthesia (PGAD/GPD). J Sex Med. 2021;18(4):665–97. 10.1016/j.jsxm.2021.01.172 33612417

[18] Grimes WR, Stratton M. Pelvic floor dysfunction. In: StatPearls [Internet]. Treasure Island (FL): StatPearls Publishing; 2025: PMID: 32644672. https://www.ncbi.nlm.nih.gov/books/NBK559246/. 32644672

[19] Kingsberg SA, Clayton AH, Pfaus JG. The female sexual response: current models, neurobiological underpinnings and agents currently approved or under investigation for the treatment of hypoactive sexual desire disorder. CNS Drugs. 2015;29:915–33. 10.1007/s40263-015-0288-1 26519340

[20] Komisaruk BR, Rodriguez del Cerro MC. Orgasm and related disorders depend on neural inhibition combined with neural excitation. Sex Med Rev. 2022;10(4):481–92. 10.1016/j.sxmr.2022.07.001 36210092

[21] Basson R. The female sexual response: A different model. J Sex Marital Ther. 2000;26(1). 10.1080/009262300278641. 10693116

[22] Terentes-Printzios D, Ioakeimidis N, Rokkas K, Vlachopoulos C. Interactions between erectile dysfunction, cardiovascular disease and cardiovascular drugs. Nat Rev Cardiol. 2022;19(1):59–74. 10.1038/s41569-021-00593-6 34331033

[23] Corona G, Maggi M. The role of testosterone in male sexual function. Rev Endocr Metab Disord. 2022;23(6):1159–72. 10.1007/s11154-022-09748-3 PMC9789013 35999483

[24] Wright LN, Moghalu OI, Das R, Horns J, Campbell A, Hotaling J, et al. Erectile dysfunction and treatment: an analysis of associated chronic health conditions. Urology. 2021;157:148–54. 10.1016/j.urology.2021.08.009 34428539

[25] McVARY K. Lower urinary tract symptoms and sexual dysfunction: epidemiology and pathophysiology. BJU Int. 2006;97:23–8. 10.1111/j.1464-410X.2006.06102.x 16507050

[26] Flegge LG, Barr A, Craner JR. Sexual functioning among adults with chronic pain: prevalence and association with pain-related outcomes. Pain Med. 2023;24(2):197–206. 10.1093/pm/pnac117 35929084

[27] Katz H, Newton-John TRO, Shires A. Sexual difficulties in the population with musculoskeletal chronic pain: a systematic review. Pain Med. 2021;22(9):1982–92. 10.1093/pm/pnaa451 33576430

[28] Hentzen C, Musco S, Amarenco G, Del Popolo G, Panicker JN. Approach and management to patients with neurological disorders reporting sexual dysfunction. Lancet Neurol. 2022;21(6):551–62. PubMed PMID: 35405093. 10.1016/S1474-4422(22)00036-9 35405093

[29] Lombardi G, Musco S, Kessler TM, Marzi VL, Lanciotti M, Del Popolo G. Management of sexual dysfunction due to central nervous system disorders: a systematic review. BJU Int. 2015;115:47–56. 10.1111/bju.13055 25599613

[30] Rees PM, Fowler CJ, Maas CP. Sexual function in men and women with neurological disorders. Lancet. 2007;369(9560):512–25. PubMed PMID: 17292771. 10.1016/S0140-6736(07)60238-4 17292771

[31] Schover LR, van der Kaaij M, van Dorst E, Creutzberg C, Huyghe E, Kiserud CE. Sexual dysfunction and infertility as late effects of cancer treatment. Eur J Cancer Suppl. 2014;12(1):41–53. 10.1016/j.ejcsup.2014.03.004 PMC4250536 26217165

[32] Goncalves MA, Guilleminault C, Ramos E, Palha A, Paiva T. Erectile dysfunction, obstructive sleep apnea syndrome and nasal CPAP treatment. Sleep Med. 2005;6(4):333–9. 10.1016/j.sleep.2005.03.001 15946896

[33] Liu Q, Zhang Y, Wang J, Li S, Cheng Y, Guo J, et al. Erectile dysfunction and depression: a systematic review and meta-analysis. J Sex Med. 2018;15(8):1073–82. 10.1016/j.jsxm.2018.05.016 29960891

[34] Liu X, Feng Z, Galling B, Qi N, Zhu X, quan, Xiao L, et al. Gender specific sexual dysfunction in patients with depression. Front Psychiatry. 2023;14:1194228. 10.3389/fpsyt.2023.1194228 PMC10309026 37398603

[35] Turan Ş, Usta Sağlam NG, Bakay H, Gökler ME. Levels of depression and anxiety, sexual functions, and affective temperaments in women with lifelong Vaginismus and their male partners. J Sex Med. 2020;17(12):2434–45. 10.1016/j.jsxm.2020.08.018 32981852

[36] Xiao Y, Xie T, Peng J, Zhou X, Long J, Yang M, et al. Factors associated with anxiety and depression in patients with erectile dysfunction: a cross-sectional study. BMC Psychol. 2023;11(1):36. 10.1186/s40359-023-01074-w PMC9899110 36739441

[37] Yang Y, Song Y, Lu Y, Xu Y, Liu L, Liu X. Associations between erectile dysfunction and psychological disorders (depression and anxiety): a cross-sectional study in a Chinese population. Andrologia. 2019;51(10):e13395. 10.1111/and.13395 31434163

[38] Yildirim E, Hacioglu Yıldırım M, Karaş H. Prevalence of depression and anxiety disorders and their relationship with sexual functions in women diagnosed with lifelong vaginismus. Turkish J Psychiatry. 2018;30. 10.5080/u22858. 31170302

[39] Atlantis E, Sullivan T. Bidirectional association between depression and sexual dysfunction: A systematic review and meta-analysis. J Sex Med. 2012;9(6). 10.1111/j.1743-6109.2012.02709.x. 22462756

[40] Atlantis E, Lange K, Goldney RD, Martin S, Haren MT, Taylor A, et al. Specific medical conditions associated with clinically significant depressive symptoms in men. Soc Psychiatry Psychiatr Epidemiol. 2011;46:1303–12. 10.1007/s00127-010-0302-3 20976591

[41] Clayton A, Kornstein S, Prakash A, Mallinckrodt C, Wohlreich M. Changes in sexual functioning associated with duloxetine, escitalopram, and placebo in the treatment of patients with major depressive disorder. J Sex Med. 2007;4(4Part1):917–29. 10.1111/j.1743-6109.2007.00520.x 17627739

[42] Atmaca M. Selective serotonin reuptake inhibitor-induced sexual dysfunction: current management perspectives. Neuropsychiatr Dis Treat. 2020;1043–50. 10.2147/NDT.S185757. PMC7182464 32368066

[43] Jing E, Straw-Wilson K. Sexual dysfunction in selective serotonin reuptake inhibitors (SSRIs) and potential solutions: A narrative literature review. Mental Health Clinician. 2016;6(4):191–6. 10.9740/mhc.2016.07.191 PMC6007725 29955469

[44] World Health Organization. Available from: https://www.who.int/news-room/fact-sheets/detail/mental-health-strengthening-our-response. 2025. Mental health: Strengthening our response.

[45] Qualitätsbericht -. Mikrozensus 2014 [Internet]. 2014 [cited 2025 Aug 18]. Available from: www.destatis.de/Kontakt.html

[46] Kronthaler SM, Tissen-Diabaté T, Karsten MM, Blohmer JU, Beier KM, Hatzler L. Assessment of Mental and Chronic Health Conditions as Determinants of Health Care Needs and Digital Innovations for Women With Sexual Dysfunction: Cross-Sectional Population-Based Survey Study in Germany. J Particip Med. 2025;17:e71301 https://jopm.jmir.org/2025/1/e71301. 2025;17(1):e71301. 10.2196/71301 PMC12386550 40865091

[47] Kronthaler SM, Hahn IJ, Schulze D, Karsten MM, Blohmer JU, Tissen-Diabaté T, et al. Sexual Distress Scale: translation, psychometric validation, and reference values for cisgender women and men in a representative population-based sample in Germany. Geburtshilfe Frauenheilkd. 2026;86(7):638–48 10.1055/a-2786-2315. PMC13372463 42459840

[48] Kronthaler SM, Tissen-Diabaté T, Karsten MM, Blohmer JU, Beier KM, Neymeyer J et al. Mental and chronic health conditions as determinants of healthcare needs and digital innovations for women with sexual dysfunction: Insights from a German population-based study. submitted for publication.

[49] Velten J, Zarski AC. Therapie-Tools Sexuelle Funktionsstörungen: Mit E-Book inside und Arbeitsmaterial. 1st ed. Weinheim: Beltz; 2022. ISBN: 978-3-621-28726-5.

[50] Derogatis LR, Revicki DA, Rosen RC, Jordan R, Lucas J, Spana C. Psychometric validation of the female sexual distress scale-desire/arousal/orgasm. J Patient Rep Outcomes. 2021;5:1–11. 10.1186/s41687-021-00359-1 PMC8463644 34559353

[51] https://www.mapi-trust.org/contact. Mapi Research Trust.

[52] Santos-Iglesias P, Mohamed B, Danko A, Walker LM. Psychometric Validation of the Female Sexual Distress Scale in Male Samples. Arch Sex Behav. 2018;47(6). 10.1007/s10508-018-1146-2. 29557994

[53] Santos-Iglesias P, Walker LM. Psychometric Validation of the Sexual Distress Scale in Men with Prostate Cancer. J Sex Med. 2018;15(7). 10.1016/j.jsxm.2018.05.015. 29960626

[54] Tavares IM, Santos-Iglesias P, Nobre PJ. Psychometric Validation of the Sexual Distress Scale in Male and Female Portuguese Samples. J Sex Med. 2022;19(5). 10.1016/j.jsxm.2022.02.026. 35370102

[55] Van Borkulo CD, Borsboom D, Epskamp S, Blanken TF, Boschloo L, Schoevers RA, et al. A new method for constructing networks from binary data. Sci Rep. 2014;4(1):5918. 10.1038/srep05918 PMC4118196 25082149

[56] Federal Statistical Office. editors. Population by age groups 2011 to 2023 in percent: Germany. https://www.destatis.de/EN/Themes/Society-Environment/Population/Current-Population/Tables/lrbev01ga.html. Accessed November 15, 2024.

[57] Bender R, Lange S. Adjusting for multiple testing–when and how? J Clin Epidemiol. 2001;54(4):343–9. 10.1016/S0895-4356(. 00)00314-0 PubMed PMID: 11297884. 11297884

[58] Epskamp S, Fried EI. A tutorial on regularized partial correlation networks. Psychol Methods. 2018;23(4):617. 10.1037/met0000167 29595293

[59] Berger MH, Messore M, Pastuszak AW, Ramasamy R. Association between infertility and sexual dysfunction in men and women. Sex Med Rev. 2016;4(4):353–65. 10.1016/j.sxmr.2016.05.002 27872029

[60] Pinheiro Sobreira Bezerra LR, Britto DF, Ribeiro Frota IP, Lira do Nascimento S, Morais Brilhante AV, Lucena SV, et al. The impact of urinary incontinence on sexual function: a systematic review. Sex Med Rev. 2020;8(3):393–402. 10.1016/j.sxmr.2019.06.009 32409182

[61] Verbeek M, Hayward L. Pelvic floor dysfunction and its effect on quality of sexual life. Sex Med Rev. 2019;7(4):559–64. 10.1016/j.sxmr.2019.05.007 31351916

[62] Liu Y, Wang Y, Pu Z, Wang Y, Zhang Y, Dong C, et al. Sexual Dysfunction in Infertile Men: A Systematic Review and Meta-Analysis. Sex Med. 2022;10(4):100528–100528. .ESXM.2022.100528 PubMed PMID: 35636279. 10.1016/j.esxm.2022.100528 PMC9386642 35636279

[63] Twitchell DK, Wittmann DA, Hotaling JM, Pastuszak AW. Psychological impacts of male sexual dysfunction in pelvic cancer survivorship. Sex Med Rev. 2019;7(4):614–26. 10.1016/j.sxmr.2019.02.003 PMC6763375 30926459

[64] Kim P, Clavijo RI. Management of male sexual dysfunction after cancer treatment. Urol Oncol. 2022;40(9):389–94. 10.1016/j.urolonc.2020.08.006. 32859462

[65] Acquati C, Hendren S, Wittmann D, Reese JB, Karam E, Duby A, et al. Psychological and sexual distress in rectal cancer patients and partners. Psychooncology. 2022;31(6):920–8. 10.1002/pon.5880 35001478

[66] Forbes MK, Baillie AJ, Schniering CA. Where do sexual dysfunctions fit into the meta-structure of psychopathology? A factor mixture analysis. Arch Sex Behav. 2016;45(8):1883–96. 10.1007/s10508-015-0613-2 26590042

[67] Forbes MK, Baillie AJ, Eaton NR, Krueger RF. A place for sexual dysfunctions in an empirical taxonomy of psychopathology. J Sex Res. 2017;54(4–5):465–85. 10.1080/00224499.2016.1269306 PMC5433908 28121167

[68] Kratzer L, Heinz P, Schennach R, Knefel M, Schiepek G, Biedermann SV, et al. Sexual symptoms in post-traumatic stress disorder following childhood sexual abuse: a network analysis. Psychol Med. 2022;52(1):90–101. 10.1017/S0033291720001750 32517829

[69] Hooten WM. Chronic pain and mental health disorders: shared neural mechanisms, epidemiology, and treatment. Mayo Clin Proc. 2016;91(7):955–70 10.1016/j.mayocp.2016.04.029. 27344405

[70] Edwards RR, Dworkin RH, Sullivan MD, Turk DC, Wasan AD. The role of psychosocial processes in the development and maintenance of chronic pain. J Pain. 2016;17(9):T70–92. 10.1016/j.jpain.2016.01.001 PMC5012303 27586832

[71] Bergman J, Gore JL, Saigal CS, Kwan L, Litwin MS. Partnership and outcomes in men with prostate cancer. Cancer. 2009;115(20):4688–94. 10.1002/cncr.24544 19626653

[72] Corona G, Mannucci E, Lotti F, Boddi V, Jannini EA, Fisher AD, et al. Impairment of couple relationship in male patients with sexual dysfunction is associated with overt hypogonadism. J Sex Med. 2009;6(9):2591–600. 10.1111/j.1743-6109.2009.01352.x 19515208

