# Supplementary material for: Links between chronic health conditions and sexual dysfunction in men: insights from a German population-based study
Source: BMC Urol. 2026 Nov 5;26:279. doi: 10.1186/s12894-026-02416-7 (PMC13737551; doi:10.1186/s12894-026-02416-7)
Supplement: Supplementary file 1 [file 12894_2026_2416_MOESM1_ESM.docx]

## Multimedia Appendix

**Links Between Chronic** **Health Conditions and Sexual Dysfunction in Men: Insights from a German Population-based Study**

Selina M Kronthaler^1*^, Carlotta F Oesterling^1*^, MSc; Tatjana Tissen-Diabaté^2^, MSc; Klaus M Beier, PhD^1^, MD; Thorsten Schlomm, MD^4^; Jörg Neymeyer, MD^4^; Laura Hatzler, MD^1,3^

^*shared first author^

^1^ Charité – Universitätsmedizin Berlin, Institute of Sexology and Sexual Medicine, Berlin, Germany

^2^ Charité – Universitätsmedizin Berlin, Institute of Social Medicine, Epidemiology and Health Economics, Berlin, Germany

^3^ Charité – Universitätsmedizin Berlin, Department of Gynecology with Breast Center, Berlin, Germany

^4^ Charité – Universitätsmedizin Berlin, Department of Urology, Berlin, Germany

**Corresponding Author:**

Laura Hatzler

Institute of Sexology and Sexual Medicine

Charité-Universitätsmedizin Berlin

Charité Platz 1, 10117 Berlin, Germany

Phone: +49 30 450 617 139

Fax:	+49 30 529 992

E-Mail: [laura.hatzler@charite.de](mailto:laura.hatzler@charite.de)

**Tables**

**Table S1.** Frequency of single SP and SD domains by CHC subgroups in n (%).

**Table S2.** Sexual Distress (FSDS-DAO) scores by CHC subgroups ranging from 0-60.

**Table S3.** Multivariable logistic regression model for SD with interaction term CHC*relationship (Model 1), in single men only (Model 2), and in partnered men only (Model 3).

**Table S4.** Weighted adjacency matrix for network A with CHC subgroups and total SP.

**Table S5.** Weighted adjacency matrix for network B with CHC subgroups and total SD.

**Table S6.** Weighted adjacency matrix for network C with CHC subgroups and individual domains of SP.

**Table S7.** Weighted adjacency matrix for network D with CHC subgroups and individual domains of SD.

**Table S8.** Threshold parameters for network models (A-D).

**Table S9.** Frequency of mental health conditions among men with comorbid mental health CHC (MH+) in n (%).

**Table S10.** Likelihood ratio test: additional variance explained by pain-related CHC in SD.

**Table S1.** Frequency of single SP and SD domains by CHC subgroups in n (%).

| **SD Domain** | **CV** | **MH** | **UR** | **IN** | **PA** | **CA** | **NE** |
| --- | --- | --- | --- | --- | --- | --- | --- |
| SP, any | 204 (77.5) | 97 (70.5) | 25 (100.0) | 37 (86.9) | 14 (72.1) | 10 (75.8) | 10 (68.5) |
| Desire problems | 159 (59.6) | 71 (52.4) | 23 (91.1) | 27 (65.4) | 12 (61.8) | 8 (62.1) | 6 (37.3) |
| Erectile problems | 146 (55.5) | 57 (41.4) | 20 (81.8) | 21 (50.0) | 9 (49.9) | 8 (66.5) | 7 (43.0) |
| Delayed ejaculation problems | 109 (41.5) | 45 (33.1) | 15 (60.3) | 14 (34.4) | 9 (50.4) | 8 (64.5) | 4 (24.3) |
| Early ejaculation problems | 114 (43.3) | 57 (41.6) | 17 (73.6) | 19 (45.1) | 7 (39.9) | 5 (42.5) | 8 (55.3) |
| SD, any | 39 (15.2) | 26 (19.2) | 12 (48.2) | 7 (16.9) | 4 (21.5) | 3 (27.1) | 1  (6.8) |
| Hypoactive sexual desire dysfunction | 16  (6.2) | 12 (8.8) | 5  (20.2) | 6 (14.2) | 4 (21.5) | 2 (14.8) | 0  (0.0) |
| Male erectile dysfunction | 28 (10.7) | 12 (9.0) | 5  (21.8) | 5 (11.6) | 4 (21.5) | 1  (8.5) | 1  (6.8) |
| Male delayed ejaculation dysfunction | 16  (6.1) | 12 (9.1) | 4  (17.0) | 4  (9.4) | 2 (11.5) | 2 (17.0) | 1  (6.4) |
| Male early ejaculation dysfunction | 23  (8.6) | 14 (10.6) | 6  (29.8) | 2  (4.4) | 2 (11.7) | 2 (17.9) | 0  (0.0) |

Note. CHC: chronic health condition(s). SD: sexual dysfunction. CV: cardiovascular and metabolic CHC. MH: mental CHC. UR: urological CHC. IN: infectious and inflammatory CHC. PA: pain-related CHC. CA: cancer CHC. NE: neurological CHC.

**Table S2.** Sexual Distress (FSDS-DAO) scores by CHC subgroups ranging from 0-60.

|  | **No**  **CHC** | **CHC** | **MH** | **CV** | **UR** | **IN** | **CA** | **PA** | **NE** |
| --- | --- | --- | --- | --- | --- | --- | --- | --- | --- |
| Median (IQR) | | | | | | | | | |
| All | 6 (0.2 - 14.0) | 12 (3.0 - 24.0) | 14 (4 - 23) | 8 (1 -19) | 17 (7 - 27) | 8 (3 - 16) | 10 (6 - 19) | 17 (4 - 39) | 10 (0 - 24) |
| In men with SP | 9 (2.0 - 17.0) | 16 (6.0 - 27.0) | 18 (7 - 26) | 13 (4 - 22) | 17 (7 - 27) | 9 (4 - 21) | 17 (7 - 19) | 27 (14 - 44) | 18 (12 - 30) |
| Mean (SD) | | | | | | | | | |
| All | 9.3 (11.1) | 14.8 (13.5) | 15.5 (13.1) | 11.5 (11.6) | 17.1 (11.5) | 10.8 (9.2) | 12.7 (12.4) | 20.8 (19.5) | 13.0 (13.9) |
| In men with SP | 12.0 (11.6) | 17.5 (13.4) | 17.9 (12.9) | 14.1 (11.8) | 17.1 (11.5) | 11.8 (9.5) | 15.2 (13.7) | 28.0 (19.3) | 19.3 (13.6) |

Abbreviation: SP, sexual problems, SD, sexual dysfunction, CHC, chronic health conditions. MH, Mental CHC. CV, Cardiovascular and metabolic CHC. UR, urological CHC. PA, pain. IN, infectious and inflammatory CHC. CA, Cancer CHC. NE, neurological CHC.

**Table S3.** Multivariable logistic regression model for SD with interaction term CHC*relationship (Model 1), in single men only (Model 2), and in partnered men only (Model 3).

|  | **Model 1**  **(n=1578)** | | | **Model 2**  **(n=577)** | | | **Model 3**  **(n=1001)** | | |
| --- | --- | --- | --- | --- | --- | --- | --- | --- | --- |
|  | OR (95% CI) | *P* value | | OR (95% CI) | *P* value | | OR (95% CI) | *P* value | |
| CHC [yes] | 4.85  (2.96 – 8.23) | | <.001 | 5.34 (3.17 – 9.34) | | <.001 | 2.68 (1.82 – 4.00) | | .001 |
| Age | 0.98  (0 0.98 – 0.99) | | <.001 | 0.99 (0.98 – 1.00) | | .075 | 0.98 (0.96 – 0.99) | | .003 |
| Sexual activity^a^ | 0.94 (0.68 – 1.29) | | .703 | 0.76 (0.50 – 1.14) | | .200 | 1.34  (0.80 – 2.21) | | .259 |
| Relationship | 1.41  (0.83 – 2.46) | | .207 |  | |  |  | |  |
| CHC* Relationship | 0.59  (0.31 – 1.10) | | .103 |  | |  |  | |  |

Abbreviation: SD, sexual dysfunction, CHC, chronic health conditions.

^a^Partnered sexual activity in the last 12 months.

**Table S4.** Weighted adjacency matrix for network A with CHC subgroups and total SP.

|  | **SP** | **MH** | **CV** | **UR** | **IN** | **CA** | **PA** | **NE** |
| --- | --- | --- | --- | --- | --- | --- | --- | --- |
| **SP** | - | 0.0 | 0.31 | 0.65 | 0.32 | 0.0 | 0.0 | 0.0 |
| **MH** | 0.0 | - | 0.0 | 0.0 | 0.0 | 0.0 | 1.03 | 0.0 |
| **CV** | 0.32 | 0.08 | - | 0.24 | 0.45 | 0.0 | 1.04 | 0.63 |
| **UR** | 0.97 | 0.0 | 0.37 | - | 0.31 | 2.22 | 1.31 | 0.75 |
| **IN** | 0.0 | 0.0 | 0.18 | 0.0 | - | 0.0 | 0.88 | 0.0 |
| **CA** | 0.0 | 0.0 | 0.0 | 2.10 | 0.0 | - | 0.0 | 0.0 |
| **PA** | 0.0 | 1.13 | 0.94 | 1.09 | 0.86 | 0.0 | - | 0.45 |
| **NE** | 0.0 | 0.0 | 0.20 | 0.0 | 0.0 | 0.0 | 0.59 | - |

Abbreviation: SP, Sexual Problems. MH, Mental CHC. CV, Cardiovascular and metabolic CHC. UR, urological CHC. PA, pain. IN, infectious and inflammatory CHC. CA, Cancer CHC. NE, neurological CHC.

**Table S5.** Weighted adjacency matrix for network B with CHC subgroups and total SD.

|  | **SD** | **MH** | **CV** | **UR** | **IN** | **CA** | **PA** | **NE** |
| --- | --- | --- | --- | --- | --- | --- | --- | --- |
| **SD** | - | 0.72 | 0.42 | 1.26 | 0.0 | 0.66 | 0.0 | 0.0 |
| **MH** | 0.50 | - | 0.0 | 0.0 | 0.17 | 0.0 | 1.13 | 0.0 |
| **CV** | 0.32 | 0.0 | - | 0.16 | 0.44 | 0.0 | 0.98 | 0.48 |
| **UR** | 0.92 | 0.0 | 0.0 | - | 0.0 | 1.64 | 1.09 | 0.0 |
| **IN** | 0.0 | 0.0 | 0.17 | 0.0 | - | 0.0 | 0.81 | 0.0 |
| **CA** | 0.47 | 0.0 | 0.0 | 1.94 | 0.0 | - | 0.0 | 0.0 |
| **PA** | 0.0 | 1.24 | 1.03 | 1.23 | 0.92 | 0.0 | - | 0.54 |
| **NE** | 0.0 | 0.0 | 0.0 | 0.0 | 0.0 | 0.0 | 0.0 | - |

Abbreviation: SD, Sexual Dysfunction. MH, Mental CHC. CV, Cardiovascular and metabolic CHC. UR, urological CHC. PA, pain. IN, infectious and inflammatory CHC. CA, Cancer CHC. NE, neurological CHC.

**Table S6.** Weighted adjacency matrix for network C with CHC subgroups and individual domains of SP.

|  | **SP1** | **SP2** | **SP3** | **SP4** | **CA** | **CV** | **NE** | **PA** | **IN** | **MH** | **UR** |
| --- | --- | --- | --- | --- | --- | --- | --- | --- | --- | --- | --- |
| **SP1** | - | 2.13 | 0.79 | 0.33 | 0.0 | 0.0 | 0.0 | 0.0 | 0.0 | 0.0 | 0.37 |
| **SP2** | 2.06 | - | 1.73 | 0.39 | 0.0 | 0.30 | 0.0 | 0.34 | 0.19 | 0.0 | 0.0 |
| **SP3** | 0.87 | 1.85 | - | 0.38 | 0.71 | 0.20 | 0.0 | 0.0 | 0.0 | 0.0 | 0.36 |
| **SP4** | 0.41 | 0.52 | 0.38 | - | 0.0 | 0.0 | 0.0 | 0.0 | 0.0 | 0.0 | 0.0 |
| **CA** | 0.0 | 0.0 | 0.70 | 0.0 | - | 0.0 | 0.0 | 0.0 | 0.0 | 0.0 | 1.94 |
| **CV** | 0.0 | 0.41 | 0.24 | 0.0 | 0.0 | - | 0.47 | 0.96 | 0.38 | 0.0 | 0.15 |
| **NE** | 0.0 | 0.0 | 0.0 | 0.0 | 0.0 | 0.0 | - | 0.0 | 0.0 | 0.0 | 0.0 |
| **PA** | 0.0 | 0.26 | 0.0 | 0.0 | 0.0 | 0.99 | 0.52 | - | 0.88 | 1.18 | 1.13 |
| **IN** | 0.0 | 0.0 | 0.0 | 0.0 | 0.0 | 0.16 | 0.0 | 0.81 | - | 0.0 | 0.0 |
| **MH** | 0.0 | 0.0 | 0.0 | 0.0 | 0.0 | 0.0 | 0.0 | 0.96 | 0.0 | - | 0.0 |
| **UR** | 0.72 | 0.0 | 0.45 | 0.0 | 1.90 | 0.21 | 0.50 | 1.24 | 0.11 | 0.0 | - |

Abbreviation: SP: sexual problems. SP1: sexual desire problems. SP2: erectile problems. SP3: delayed ejaculation problems. SP4: early ejaculation problems. MH: mental CHC. CV: cardiovascular and metabolic CHC. UR: urological CHC. IN: infectious and inflammatory CHC. CA: cancer CHC. PA: pain-related CHC. NE: neurological CHC.

**Table S7.** Weighted adjacency matrix for network D with CHC subgroups and individual domains of SD.

|  | **SD1** | **SD2** | **SD3** | **SD4** | **CA** | **CV** | **NE** | **PA** | **IN** | **MH** | **UR** |
| --- | --- | --- | --- | --- | --- | --- | --- | --- | --- | --- | --- |
| **SD1** | - | 2.74 | 1.45 | 0.90 | 0.0 | 0.0 | 0.0 | 0.0 | 0.0 | 0.0 | 0.0 |
| **SD2** | 2.60 | - | 2.83 | 0.65 | 0.0 | 0.44 | 0.0 | 0.0 | 0.0 | 0.31 | 0.0 |
| **SD3** | 1.58 | 3.01 | - | 0.75 | 0.71 | 0.0 | 0.0 | 0.0 | 0.0 | 0.0 | 0.0 |
| **SD4** | 1.16 | 0.93 | 0.78 | - | 0.0 | 0.0 | 0.0 | 0.0 | 0.0 | 0.0 | 0.0 |
| **CA** | 0.0 | 0.0 | 0.77 | 0.0 | - | 0.0 | 0.0 | 0.0 | 0.0 | 0.0 | 1.88 |
| **CV** | 0.0 | 0.69 | 0.0 | 0.0 | 0.0 | - | 0.50 | 0.97 | 0.42 | 0.0 | 0.16 |
| **NE** | 0.0 | 0.0 | 0.0 | 0.0 | 0.0 | 0.0 | - | 0.0 | 0.0 | 0.0 | 0.0 |
| **PA** | 0.0 | 0.0 | 0.0 | 0.0 | 0.0 | 0.99 | 0.50 | - | 0.91 | 1.16 | 1.18 |
| **IN** | 0.0 | 0.0 | 0.0 | 0.0 | 0.0 | 0.19 | 0.0 | 0.88 | - | 0.0 | 0.0 |
| **MH** | 0.0 | 0.41 | 0.0 | 0.0 | 0.0 | 0.0 | 0.0 | 0.97 | 0.0 | - | 0.0 |
| **UR** | 0.48 | 0.25 | 0.30 | 0.16 | 1.59 | 0.0 | 0.0 | 1.10 | 0.0 | 0.0 | - |

Abbreviation: SD: sexual dysfunction. SD1: hypoactive sexual desire dysfunction (HSDD). SD2: male erectile dysfunction (ED). SD3: male delayed ejaculation dysfunction (DE). SD4: Male early ejaculation dysfunction (PE). MH: mental CHC. CV: cardiovascular and metabolic CHC. UR: urological CHC. IN: infectious and inflammatory CHC. CA: cancer CHC. PA: pain-related CHC. NE: neurological CHC.

**Table S8.** Threshold parameters for network models (A-D).

|  | **Threshold** | | | |
| --- | --- | --- | --- | --- |
| **Symptom** | **Network A** | **Network B** | **Network C** | **Network D** |
| SP | 0.85 |  |  |  |
| SP1 |  |  | -1.15 |  |
| SP2 |  |  | -2.25 |  |
| SP3 |  |  | -2.44 |  |
| SP4 |  |  | -0.96 |  |
| SD |  | -2.12 |  |  |
| SD1 |  |  |  | -3.68 |
| SD2 |  |  |  | -3.62 |
| SD3 |  |  |  | -3.94 |
| SD4 |  |  |  | -2.91 |
| MH | -1.45 | -1.58 | -1.44 | -1.49 |
| CV | -1.01 | -0.79 | -1.01 | -0.80 |
| UR | -3.94 | -2.98 | -3.64 | -2.91 |
| IN | -2.29 | -2.29 | -2.30 | -2.33 |
| CA | -3.47 | -3.54 | -3.77 | -3.53 |
| PA | 3.18 | -3.32 | -3.39 | -3.25 |
| NE | -3.31 | -3.14 | -3.13 | -3.14 |

Abbreviation: SP: sexual problems. SP1: sexual desire problems. SP2: erectile problems. SP3: delayed ejaculation problems. SP4: early ejaculation problems. SD: sexual dysfunction. SD1: hypoactive sexual desire dysfunction (HSDD). SD2: male erectile dysfunction (ED). SD3: male delayed ejaculation dysfunction (DE). SD4: Male early ejaculation dysfunction (PE). MH: mental CHC. CV: cardiovascular and metabolic CHC. UR: urological CHC. IN: infectious and inflammatory CHC. CA: cancer CHC. PA: pain-related CHC. NE: neurological CHC.

**Table S9.** Frequency of mental health conditions among men with comorbid mental health CHC (MH+) in n (%).

| **Mental health condition** | **n** | **% of MH+ group** | **% of total sample** |
| --- | --- | --- | --- |
| Depression | 244 | 73.7 | 14.0 |
| Anxiety disorders | 134 | 40.5 | 7.7 |
| Post-traumatic stress disorder | 41 | 12.4 | 2.4 |
| Autism | 6 | 1.8 | 0.3 |
| Other mental health conditions | 142 | 42.9 | 8.2 |

Note. MH+: CHC with at least one comorbid mental health condition. Percentages may exceed 100% as participants could report more than one mental health condition. Total MH+ group: n = 331.

**Table S10.** Likelihood ratio test: additional variance explained by pain-related CHC in SD.

| **Model** | **Resid_Df** | **Resid_Dev** | **Df** | **Deviance** | **F** | **p** |
| --- | --- | --- | --- | --- | --- | --- |
| Without pain | 1383 | 1178 | - | - | - | - |
| With pain | 1382 | 1178 | 1 | 0.026 | 0.03 | 0.87 |

Note. Model 1: SD regressed on age, partnered sexual activity, relationship status, and comorbid CHC subgroups (CV+, MH+, UR+, IN+, CA+, NE+). Model 2: Model 1 with additional inclusion of pain-related CHC (PA+). An F-test was used to assess whether PA+ explained significant additional variance in SD over and above the other CHC subgroups. PA+: comorbid pain-related CHC. CV+: comorbid cardiovascular and metabolic CHC. MH+: comorbid mental health CHC. UR+: comorbid urological CHC. IN+: comorbid infectious and inflammatory CHC. CA+: comorbid cancer-related CHC. NE+: comorbid neurological CHC.
